# Fully phased human genome assembly without parental data using single-cell strand sequencing and long reads

**DOI:** 10.1038/s41587-020-0719-5

**Published:** 2020-12-07

**Authors:** David Porubsky, Peter Ebert, Peter A. Audano, Mitchell R. Vollger, William T. Harvey, Pierre Marijon, Jana Ebler, Katherine M. Munson, Melanie Sorensen, Arvis Sulovari, Marina Haukness, Maryam Ghareghani, Human Genome Structural Variation Consortium, Peter M. Lansdorp, Benedict Paten, Scott E. Devine, Ashley D. Sanders, Charles Lee, Mark J. P. Chaisson, Jan O. Korbel, Evan E. Eichler, Tobias Marschall

**Affiliations:** 1 Department of Genome Sciences, University of Washington School of Medicine, Seattle, WA USA; 2 Heinrich Heine University Düsseldorf, Medical Faculty, Institute for Medical Biometry and Bioinformatics, Düsseldorf, Germany; 3 UC Santa Cruz Genomics Institute, University of California, Santa Cruz, Santa Cruz, CA USA; 4 Center for Bioinformatics, Saarland University, and Max Planck Institute for Informatics, Saarbrücken, Germany; 5 Terry Fox Laboratory, BC Cancer Agency, Vancouver, British Columbia Canada; 6 Department of Medical Genetics, University of British Columbia, Vancouver, British Columbia Canada; 7 Institute for Genome Sciences, University of Maryland School of Medicine, Baltimore, MD USA; 8 European Molecular Biology Laboratory, Genome Biology Unit, Heidelberg, Germany; 9 The Jackson Laboratory for Genomic Medicine, Farmington, CT USA; 10 The First Affiliated Hospital of Xi’an Jiaotong University, Xi’an, China; 11 Department of Life Science, Ewha Womans University, Seoul, Republic of Korea; 12 Quantitative and Computational Biology, University of Southern California, Los Angeles, CA USA; 13 Howard Hughes Medical Institute, University of Washington, Seattle, WA USA

**Keywords:** Bioinformatics, Genomic analysis, Sequencing, Genome informatics

## Abstract

Human genomes are typically assembled as consensus sequences that lack information on parental haplotypes. Here we describe a reference-free workflow for diploid de novo genome assembly that combines the chromosome-wide phasing and scaffolding capabilities of single-cell strand sequencing^1,2^ with continuous long-read or high-fidelity^3^ sequencing data. Employing this strategy, we produced a completely phased de novo genome assembly for each haplotype of an individual of Puerto Rican descent (HG00733) in the absence of parental data. The assemblies are accurate (quality value > 40) and highly contiguous (contig N50 > 23 Mbp) with low switch error rates (0.17%), providing fully phased single-nucleotide variants, indels and structural variants. A comparison of Oxford Nanopore Technologies and Pacific Biosciences phased assemblies identified 154 regions that are preferential sites of contig breaks, irrespective of sequencing technology or phasing algorithms.

## Abstract (web summary)

Assembly of haplotype-resolved human genomes is achieved by combining short and long reads.

## Main

The first attempt to assemble a diploid human genome from a single individual relied on highly accurate and moderately long (500–1,000-bp) Sanger sequencing reads^4^. However, such assemblies were fragmented and unable to resolve many repetitive regions of the human genome^4^. Recent advances in long-read sequencing technologies (led by Pacific Biosciences (PacBio) and Oxford Nanopore Technologies (ONT)) allow the generation of accurate and much more contiguous genome assemblies. By circumventing the problem of haplotype separation through sequencing of fully homozygous hydatidiform mole cell lines^5,6^, one can achieve highly contiguous assemblies, which, in some instances, traverse centromeric regions^7^. For diploid samples, haplotype separation has been demonstrated using long reads^8^ or linked reads^9^ (phased block N50: 169–277 kbp); but such approaches lack global phase information and are, thus, unable to separate haplotypes over extended genomic distances. Global haplotype partitioning of reads before assembly was achieved using sequencing data of the parents in conjunction with long reads—for example, by leveraging parent-specific *k*-mers.^10^ However, such parental sequencing data are not always available, especially in clinical settings. A promising direction for obtaining single-individual phased assemblies combines long reads with Hi-C data^11,12^, but reliable scaffolding and phasing across entire chromosomes remain challenging.

**Fig. 1:**
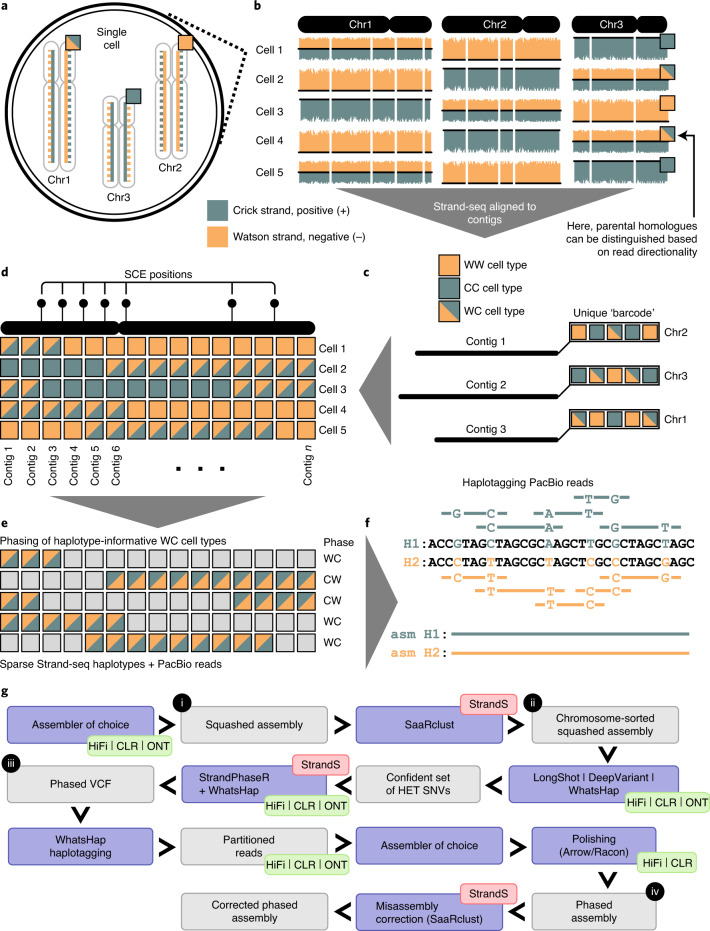
Overview of the genome assembly pipeline. **a**, In a single Strand-seq library, only the template DNA strand (solid line) is sequenced for each parental homologous chromosome. **b**, Template strands of each homologue in a given diploid cell are randomly inherited by daughter cells (‘+’ positive strand, teal—Crick and ‘−’ negative strand, orange—Watson), resulting in three possible template strand states for homologous chromosomes (height of bars plotted along each chromosome represents the number of ‘+’ and ‘−’ reads mapped in each genomic bin): WC, one Crick and one Watson strand represented for given homologues; WW, only Watson template strands represented; or CC, only Crick template strands represented. **c**, Unassigned contigs follow the same pattern of template strand state inheritance based on the homologue they belong to. **d**, Contig order can be inferred based on low-frequency changes in a template strand state resulting from sister chromatid exchange (SCE) events in the parental cell: contigs that are closer to each other tend to share the same template strand state more often than more distant contigs. **e**, Regions with WC strand state are haplotype informative and can be assembled into continuous haplotypes. **f**, Haplotypes can then be used to split long reads into their respective homologues. **g**, Generation of long-read (HiFi/CLR/ONT)-based assemblies: 1) producing squashed assemblies; 2) assigning contigs to clusters using Strand-seq (StrandS); 3) phasing clustered assemblies using the combination of Strand-seq and long PacBio reads; and 4) partitioning and reassembling of haplotype-specific PacBio reads and polishing of the final diploid assemblies.

Strand sequencing (Strand-seq) is a short-read, single-cell sequencing method that preserves structural contiguity of individual homologs in every single cell (Fig. 1a). This is achieved by using a thymidine analog to selectively label and remove one of the DNA strands (the nascent strand, synthesized during DNA replication), which generates directional sequencing libraries of DNA template strands only (Supplementary Notes)^1,2^. Strand-seq has three important abilities: 1) it can sort reads or contigs by chromosome^13–16^; 2) it can order and orient contigs^13^; and 3) it provides a chromosome-wide phase signal irrespective of physical distance^17^. These features make Strand-seq an ideal method to be combined with long-read sequencing data to physically phase^18^ and assemble diploid genomes. Previously, we used this approach for partitioning reads before local assembly to improve structural variation sensitivity^19^, but read partitioning required mapping to a reference genome as an intermediate step. Here we show how this limitation can be removed by exploiting Strand-seq’s additional ability to assign contigs to chromosomes to phase them and how this linking technology can be coupled with long-read sequencing (continuous long-read (CLR), high-fidelity (HiFi) or ONT). We present a completely reference-free workflow for diploid genome assembly and demonstrate accurate assembly of parental haplotypes of a ~6-Gbp genome.

Our unified assembly workflow starts by producing haplotype-unaware (‘squashed’) de novo assemblies from the full set of long reads from both haplotypes. We then align Strand-seq data to the contigs resulting from the de novo assembly (Fig. 1b). We use the SaaRclust package^15^, extended here to work with raw contigs (Supplementary Notes), to assign each contig to a unique cluster. Each cluster is defined by a unique strand inheritance over multiple Strand-seq libraries and ideally represents a single chromosome (Fig. 1c and Supplementary Notes). Furthermore, we infer the order of contigs within each cluster (chromosome) by leveraging sister chromatid exchange (SCE) events identified with Strand-seq (Fig. 1d)^1,20,21^. This clustering by chromosome is a key step that enables haplotype phasing. To this end, we align both long sequencing reads and Strand-seq data back to the clustered assemblies. Our assembly pipeline next calls heterozygous (HET) single-nucleotide variants (SNVs) using the long reads to obtain a confident set of markers for phasing. We use these heterozygous SNVs to reconstruct global chromosome-length haplotypes using WhatsHap^22,23^, combining Strand-seq and PacBio reads (Fig. 1e)^18^. The resulting phased SNVs are then used to tag and split long reads per haplotype, again using WhatsHap (Fig. 1f). For each set of haplotype-specific reads, our workflow performs a complete de novo assembly of each parental homolog, alternatively using wtdbg2 (ref. ^24^), Flye^25^, Canu^26^ or Peregrine^27^, and polishes the assemblies twice with Racon^28^ to obtain the final diploid assemblies (Fig. 1g).

**Fig. 2:**
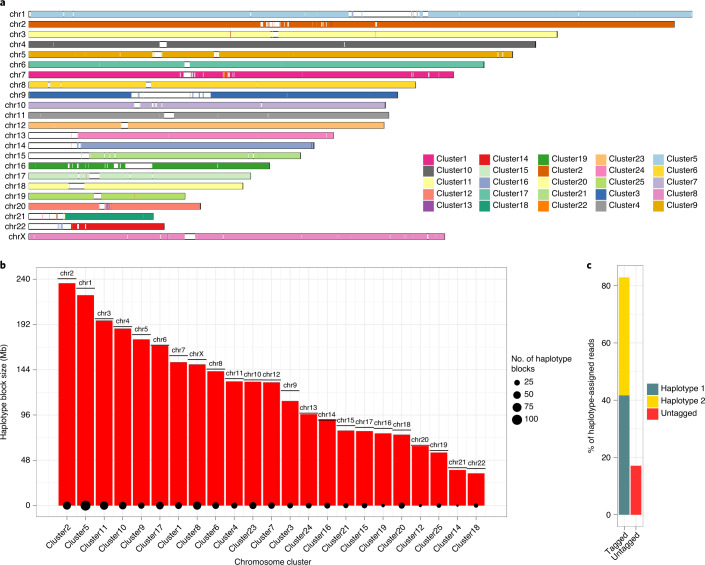
Reference-free scaffolding and phasing of squashed assembly for HG00733. **a**, Each contig represents a range based on mapping coordinates on GRCh38. Contigs are colored based on cluster identity determined by SaaRclust. In an ideal scenario, there is a single color for each chromosome. **b**, The size of the longest haplotype block per chromosome is shown as red bars, with the remaining haplotype blocks of negligible length. The size of the point at the bottom of each bar reflects the number of haplotype blocks in each cluster. For perspective, the real size of each chromosome for GRCh38 is plotted as a horizontal solid line. **c**, The percentage of PacBio reads successfully assigned to either H1 (teal) or H2 (yellow). Reads that could not be assigned to either haplotype are shown in red.

To demonstrate the utility of our workflow for building a completely phased genome assembly, we generated ~33.4-fold HiFi sequence coverage from a single individual (HG00733) of Puerto Rican descent from the 1000 Genomes Project^29^ as well as ~32-fold and ~21-fold coverage of HiFi reads of the parental genomes (HG00731 and HG00732) for validation purposes, respectively. We initially assembled HiFi reads for HG00733 using Peregrine^27^ into a squashed assembly with contig N50 of ~34 Mbp. To scaffold the genome, we aligned 115 single-cell Strand-seq libraries generated for HG00733 (ref. ^19^) to the squashed assembly contigs. The cumulative depth of Strand-seq reads was 2.87-fold and covered 73% of genomic positions in the assembly. After clustering these contigs by chromosomes using SaaRclust, we aligned all contigs back to GRCh38 for evaluation purposes. Overall, 86.4% mapped back to their respective chromosome of origin, with the bulk of misassignments corresponding to small contigs (median size, 139,157 bp). Notably, 99.8% of the total length of all clustered contigs were assigned to their correct chromosomal origin (Fig. 2a). The high accuracy of our chromosomal scaffolds is supported by independent proximity ligation (Hi-C) data (Supplementary Fig. 1a).

Using DeepVariant, we detected 2,487,405 heterozygous SNVs genome wide within the squashed assembly. Phasing these variants using the Strand-seq signal and the HiFi reads^18^ resulted in chromosome-length haplotypes with more than 99% (Supplementary Fig. 1b, red line) of all these heterozygous variants placed into a single haplotype block. The longest haplotype block spanned almost the entire length of each cluster (Fig. 2b, red bars) and closely matched the expected chromosome lengths from GRCh38 (Fig. 2b, solid horizontal lines, and Supplementary Fig. 2). With such global and complete haplotypes, we assigned ~83% of the original HiFi reads to either parental haplotype 1 (H1) or haplotype 2 (H2) (Fig. 2c). The remaining ~17% of haplotype-unassigned reads likely originate from autozygous regions and low-mappability regions, such as segmental duplications (SDs) and heterochromatic regions. To find the minimum number of Strand-seq libraries required to produce phased assemblies, we downsampled the original number of Strand-seq libraries (*n* = 115). We found that 40% of the libraries are sufficient to correctly cluster contigs into chromosomal scaffolds (Supplementary Fig. 3) and to phase more than 82% of HiFi reads (Supplementary Fig. 4).

We next assembled haplotype-specific reads into completely phased de novo assemblies using Peregrine^27^, resulting in highly contiguous assemblies (N50 contig: H1, 23.7 Mbp; H2, 25.9 Mbp) (Supplementary Table 1). By assembling reads per cluster, we effectively avoid creation of chimeric contigs (Supplementary Fig. 5a), whereas the residual assembly errors (misorientations) can be readily identified and corrected by SaaRclust (Supplementary Fig. 6 and Methods). We found that most (~83%) of misassemblies made by Peregrine were in the vicinity of SDs of size 50 kbp and longer (Supplementary Fig. 5b). This is expected as high-identity SDs promote false joins during the assembly process^30^. After assembly error correction, we were left with a total of four misorientations (in contigs ≥1 Mbp) that reside at the very ends of affected contigs (Supplementary Notes).

Our pipeline is also able to process long error-prone reads such PacBio CLR or ONT reads. The resulting phased assemblies were of remarkable contiguity for both CLR (N50 contig: H1, 24 Mbp; H2, 23.5 Mbp) and ONT (N50 contig: H1, 33.4 Mbp; H2, 36.2 Mbp) reads (Supplementary Table 1 and Supplementary Fig. 7). For comparison purposes, we also ran our assembly pipeline on the HiFi datasets of the two parents, yielding assemblies that were slightly less continuous due to the lower input coverage (contig N50: HG00731: H1, 19.9 Mbp; H2, 20.1 Mbp; HG00732: H1, 10.4 Mbp; H2, 10.8 Mbp) (Supplementary Table 1). To verify the ability to also process nonhuman data^31^, we clustered squashed contigs from a gorilla PacBio assembly and correctly assigned contigs to 24 clusters while, at same time, resolving known reciprocal translocations between chromosomes 5 and 17 (in humans) (Supplementary Fig. 8).

After phased assembly, we used Strand-seq data to assign Peregrine contigs (HG00733, HiFi) into whole-chromosomal scaffolds. First, we assigned each contig (≥500 kbp) to its chromosome of origin (Supplementary Fig. 9a), with more than 99.9% of a total contig length correctly assigned for both haplotype assemblies. Second, we synchronized the orientation of all contigs within each chromosomal scaffold in both haplotypes. Notably, after the contig reorientation process, 99.5% and 99.7% of a total contig length mapped to GRCh38 in a single direction for H1 and H2, respectively (Supplementary Fig. 9b). Lastly, we ordered contigs within both phased assemblies, obtaining an ordering that highly correlated (mean Pearson correlation: H1, 0.94; H2, 0.947) with the expected contig order (Supplementary Fig. 9c and Supplementary Fig. 10).

**Fig. 3:**
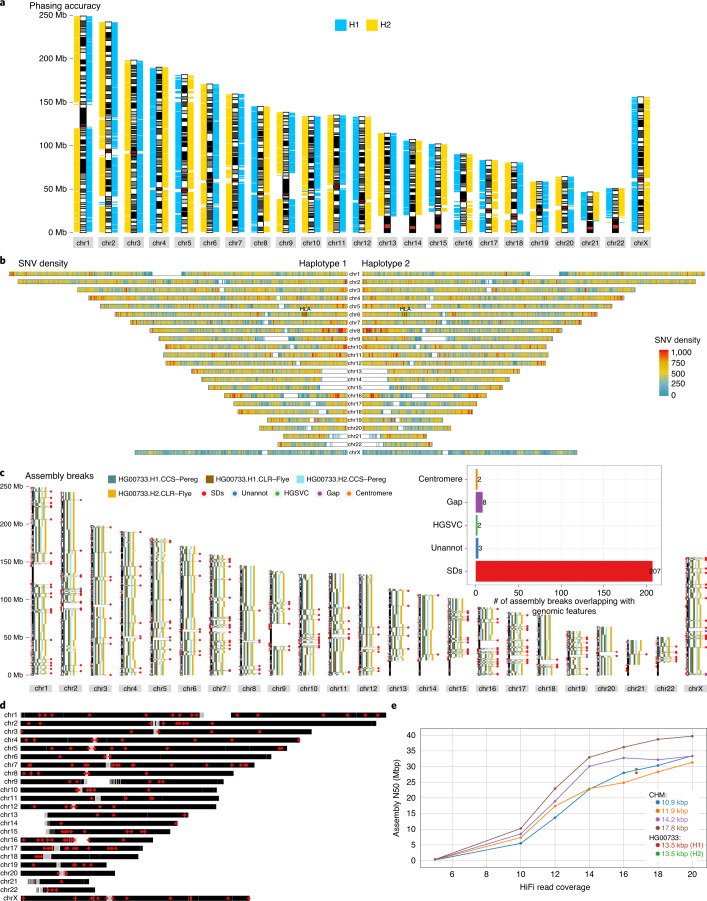
Phased assembly analysis and common assembly breaks. **a**, Each 1-Mbp block of phased contigs (Freeze 1.1; ‘Data availability’) are assigned to one of the parental genomes using SNV data from the parents^19^: maternal segments (HG00732) are shown in blue; paternal segments (HG00731) are shown in yellow. **b**, Genome-wide summary of SNV density counted in 500-kbp genomic bins sliding by 10 kbp. The HLA locus on chromosome 6 is labeled as ‘HLA’. **c**, An ideogram shows aligned contigs separately for H1 and H2. Subsequent contigs are plotted as discontiguous rectangles along each chromosome. Positions of common breaks (*n* = 222) between Flye (CLR reads) and Peregrine (HiFi reads) assemblies are highlighted by horizontal lines and their overlap with various genomic features, such as SDs, is marked by colored dots. Note: owing to the difficulty of aligning contigs continuously over the centromeres, we flag these regions as unresolved. Inset, a bar plot summarizing the total counts for each genomic feature across all 222 assembly breaks. Unannot, unannotated assembly breaks. **d**, An ideogram shows genomic positions of 154 common assembly breaks shared by multiple assembles. Gray rectangles represent centromeric positions, whereas white rectangles point to genome gaps. **e**, Effect of coverage and read length on assembly contiguity. Points connected by lines represent the N50s of Peregrine assemblies for CHM libraries as a function of coverage (blue, CHM13, 10.9 kbp; orange, CHM1, 11.9 kbp; purple, CHM13, 14.2 kbp; brown, CHM13; 17.8 kbp). These assemblies show what contiguity is attainable with Peregrine given different read lengths and coverages in a genome with only one haplotype. Highlighted in red and green are the two Peregrine assemblies of the haplotypes of HG00733 (red, H1, 13.5 kbp; green, H2, 13.5 kbp).

To confirm that the haplotype-resolved genome assemblies were correctly phased across all chromosomes, we independently assigned each 1-Mbp window of the assembled contigs to one of the two parents (that is, HG00731 and HG00732; Methods) by using a set of trio-phased SNVs produced earlier^19^. As expected, the child (HG00733) assembly was correctly phased, with only sporadic local errors (Fig. 3a) amounting to a switch error rate of ~0.17% and a Hamming distance of ~0.17%. To specifically assess phasing performance at a challenging but biomedically relevant locus, we examined the whole major histocompatibility complex (MHC) region and found that it was traversed by a single contig in both haplotype assemblies. These phased assemblies were consistent with recently released Shasta assemblies^32^ that used trio-binned ONT data, with a Hamming error rate of 0.28% (Methods and Supplementary Fig. 11), and represented some of the most diverse regions of the genome (Fig. 3b).

We generated estimates of the consensus quality value (QV) of our assembly using several independent methods. We sequenced and assembled 78 random bacterial artificial chromosomes (BACs) from an HG00733 clone library (VMRC62) and compared these sequences to the phased assemblies to estimate the consensus QV (Methods). We found the median BAC-based QV to be 40.47, which corresponds to less than one error every 10,000 bases. Additionally, we derived QV estimates based on variant callsets generated by mapping Illumina short reads to the assemblies. By identifying homozygous calls within high-confidence regions (Methods), we computed QV estimates reaching an upper bound of 60 (Supplementary Table 1 and Methods). Overall, our QV estimates are similar to the QV achieved in the HiFi assembly of a haploid human genome, CHM13 (for example, BAC QV 40.47 versus 45.25)^33^. Despite the lower coverage per phased haplotype, we were able to resolve a similar level of SDs on both haplotypes. We estimate that 32.13% and 32.31% of SDs were resolved in the H1 and H2 assemblies of HG0733, respectively (Methods). This estimate is similar to Peregrine assemblies of CHM13 assembled with 16- and 18-fold coverage—both of which resolved an estimated 35.8% of SDs. The H1 and H2 assemblies showed signs of increased read coverage over 22.4 Mbp and 22.0 Mbp of their respective assemblies^34^ (Methods), indicating the presence of collapsed SDs or other repetitive sequences. Of these regions, 120 (H1) and 126 (H2) correspond to collapsed duplications longer than 50 kbp. As a final measure of quality control, we performed a joint comparative analysis of the assemblies of the two parents and the child assemblies produced from HiFi, CLR and ONT data, showing that 99.2% of the genotypes derived from the HiFi assemblies had orthogonal support from either CLR or ONT assemblies or from displaying Mendelian consistency (Supplementary Note).

To compare our assemblies with proximity ligation–based (Hi-C) assemblies by Garg et al.^12^, we used public Strand-seq and HiFi data to create a phased assembly of NA12878 (contig N50 H1, 18.3 Mbp; H2, 21.9 Mbp) (Supplementary Table 1 and ‘Data availability’). In comparison to Garg et al., we are able to correct most misassemblies produced by Peregrine (Supplementary Table 2 and Supplementary Fig. 12) and synchronize directionality of contigs within each chromosomal scaffold with more than 99.5% accuracy (Supplementary Fig. 13). We achieved better phasing accuracy of the final phased assemblies with very low Hamming distance (~0.4%) and switch error rate (~0.45%) (Supplementary Table 2, Supplementary Fig. 14 and Methods). Lastly, we emphasize the robustness of clustering contigs by chromosome using Strand-seq (Supplementary Fig. 2), whereas scaffolding from Hi-C can lead to less robust results (Supplementary Fig. 15). The good performance of our assembly pipeline was confirmed also in comparison to FALCON-phase^11^ assemblies (Supplementary Table 2).

To discover genetic variation, we aligned contigs from both haplotypes of the HG00733 HiFi assemblies to GRCh38 and identified SNVs, indels and structural variants (SVs) based on a previously described approach^30^, which were then merged to create a set of heterozygous and homozygous calls (Methods). We identified a total of 4.1 million SNVs (~2.8 million per haplotype) (Fig. 3b) and 1.01 million indels distributed among insertions and deletions (515,687 and 497,067, respectively) (Supplementary Table 3 and Supplementary Fig. 16a). Regions of increased genetic diversity were observed near the telomeres and human leukocyte antigen (HLA) genes, as expected (Fig. 3b and Supplementary Fig. 16b,c). In contrast, we also observed five extended regions of loss of heterozygosity that are not due to deletion (Supplementary Fig. 17 and Methods). In addition, we identified SVs including 15,093 insertions and 9,519 deletions (Supplementary Table 4 and Supplementary Fig. 18). Considering gene-disruptive indels and SVs, we observed 223 disrupted genes in our diploid genome compared to 135 per haploid genome^33^ (Supplementary Table 5). If we exclude repetitive regions, where variants are often difficult to compare because of alignment issues, and use Human Genome Structural Variation Consortium (HGSVC) HG00733 calls^19^ as a truth set, we estimate 92% sensitivity and 92% specificity (Supplementary Fig. 19). If we include repetitive regions, we estimate 65% sensitivity and 73% specificity, mostly due to a difficulty in comparing variant calls in tandem repeat (TR) sequences (Supplementary Fig. 20). Lastly, we used the six haplotype assemblies of the whole trio (HG00731, HG00732 and HG00733) to identify 49 and 65 meiotic recombination breakpoints in the paternal and maternal homologs of HG00733, respectively. We found 92.7% and 89.3% of previously identified meiotic recombination breakpoints^19^ to be within 1 kbp from the breakpoints detected in our phased assemblies (Supplementary Fig. 21a). As expected, we found more male meiotic breakpoints (*n* = 9) within 5-Mbp distance from telomeres than female (*n* = 4) (Supplementary Fig. 21b).

There are regions of the genome that have been notoriously difficult to assemble, even with long-read technologies^6,35^. In this study, we operationally defined such difficult regions of the human genome as positions where both phased assemblies, made by Peregrine (HiFi data) and Flye (CLR data), consistently break. In total, we localized 222 common breaks in our phased de novo assemblies (Fig. 3c). The vast majority (93%) of these assembly breakpoints lie within SD-rich regions of the genome that are copy number variable (*P* < 0.0001; mean enrichment, ~eight-fold) (Supplementary Fig. 22a), many of which are more than 50 kbp in length and are highly repetitive (Supplementary Fig. 22b). This results in an extremely interconnected assembly graph that is difficult to resolve (Supplementary Fig. 22c). To determine whether these 222 common assembly breaks are shared among other phased assemblies, we examined a recently released Shasta ONT assembly of the same individual^32^. We found that 154 of those breaks disrupt the Shasta assembly as well (Fig. 3d and Supplementary Table 6), and 110 of these regions overlap SVs detected by the HGSVC, of which 65 overlap with inversions (Supplementary Fig. 22d). Even the most contiguous assembly of a haploid genome (CHM13) to date^7^, constructed from ultra-long ONT reads and PacBio data, shares 64 common assembly breaks. We propose that these universal assembly breaks (UABs) represent regions of our genome where neither the sequencing technology nor assembly algorithms can resolve the underlying sequence in an automated fashion. These UAB regions represent compositional features of the human genome and not the result of incomplete phasing of long-read data. For example, even when sequence reads are fully phased (as in the case of haploid genomes), increasing coverage and insert size only moderately improves contiguity (Fig. 3e), and the two human genomes we assembled here have reached that empirical upper bound based on comparisons to human haploid references^33^.

In summary, we introduced an assembly workflow to combine Strand-seq and long reads (PacBio or ONT) in a completely reference-free manner to provide fully phased and highly contiguous de novo assemblies of diploid human genomes. Previously, this was possible only by resorting to parental genome sequencing. Our assembly strategies allow us to transition from squashed human assemblies of ~3 Gbp to fully phased assemblies of ~6 Gbp where all types of genetic variants, including SVs, are fully phased at the haplotype level. We provide evidence that our workflow produces high-quality assemblies in a robust manner by assembling the Puerto Rican individual HG00733 with three different long-read sequencing datasets (PacBio HiFi/circular consensus sequencing (CCS), CLR and ONT). Our pipeline is designed for seamlessly switching between software tools for the individual tasks—for example, Flye^25^, Shasta^32^, wtdbg2 (ref. ^24^), Peregrine^27^ and Canu^26^ can be used for (haploid) assembly, and FreeBayes^36^, LongShot^37^, DeepVariant^38^ and WhatsHap genotyping^39^ can be used for variant calling. This method should open the door for producing high-quality phased human genomes needed for personalized SV discovery in healthy and diseased individuals. Fully phased, reference-free genomes are also the first step in constructing comprehensive human pangenome references that aim to reflect the full range of human genome variation^40^. Our work also highlights recalcitrant regions of genome assembly that will require further advances in sequencing technology and algorithms.

## Methods

### Cell lines

Cell lines for Puerto Rican individuals HG00731, HG00732 and HG00733 have been previously described^19^.

### HiFi PacBio sequencing

Isolated DNA was prepared for HiFi library preparation as described^3^. Briefly, DNA was sheared to an average size of about 15 kbp using Covaris gTUBE, and the quantity and size were checked using Qubit (Thermo Fisher) and FEMTO Pulse (Agilent) instruments. Fragments underwent library preparation using the Template Prep Kit v1 (PacBio) and then fractionation on a SageELF (Sage Science) instrument. After evaluating size, fractions averaging 11, 13 or 15 kbp were sequenced on a Sequel II (PacBio) instrument using Sequel II chemistry v1 or v2EA (Early Access beta). After sequencing, raw data were analyzed with SMRT Link 7.1 or 8.0 using the CCS protocol with a cutoff minimum of three passes and estimated accuracy of 0.99. In total, 18 SMRT Cell 8Ms were run for the Puerto Rican trio (HG00731, HG00732 and HG00733) for an average yield per sample of 91 Gbp of HiFi reads (Supplementary Table 7).

### Strand-seq data analysis

All Strand-seq data in a FASTQ format were obtained from publicly available sources (‘Data availability’). At every step that requires alignment of short-read Strand-seq data to the squashed or clustered de novo assembly (Fig. 1), we used BWA-MEM (version 0.7.15-r1140) with the default parameters. In the next step, we filtered out all secondary and supplementary alignments using SAMtools (version 1.9). Subsequently, duplicate reads were marked using Sambamba (version 0.6.8). For every Strand-seq data analysis, we filtered out reads with mapping quality less than 10 as well as all duplicate reads.

### Squashed genome assembly

Initially, squashed assemblies were constructed to produce a set of unphased contigs. We assembled HiFi reads using the Peregrine assembler.

All Peregrine (v0.1.5.5) assemblies were run using the following command:

pg_run.py asm {reads.fofn} 36 36 36 36 36 36 36 36 36 --with-consensus \--shimmer-r 3 --best_n_ovlp 8 --output {assembly.dir}

### Clustering contigs into chromosomal scaffolds

We used the R package SaaRclust^15^ to cluster de novo squashed assemblies into chromosomal scaffolds. SaaRclust takes as an input Strand-seq reads aligned to the squashed de novo assembly in a BAM format. Given the parameter settings, we discarded contigs shorter than 100 kbp from further analysis. Remaining contigs were partitioned into variable sized bins of 200,000 Strand-seq mappable positions. The counts of aligned reads per bin, separated by directionality (+/Crick or −/Watson), are used as an input for SaaRclust that divides contigs into a user-defined number of clusters (set to *n* = 100|150). Contigs genotyped as Watson–Crick (WC) in most cells were discarded. We further removed contigs that could be assigned to multiple clusters with probability *P* < 0.25 (Supplementary Fig. 23). Subsequently, SaaRclust merges clusters that share the same strand inheritance across multiple Strand-seq libraries. Shared strand inheritance is used to construct a graph of connected components (clusters), and the most connected subgraphs are reported, resulting in approximately 24 clusters—that is, one cluster should ideally be representative of one human chromosome. Next, we defined misoriented contigs within each cluster as those having opposing directionality in every Strand-seq library. We used hierarchical clustering to detect groups of minus-oriented and plus-oriented contigs. To synchronize contig directionality, we switch direction in one group of contigs from plus to minus or vice versa. Contigs synchronized by direction are then subjected to positional ordering within a cluster. We again use contig strand state coinheritance as a proxy to infer physical distance for each contig pair in every Strand-seq library. The resultant coinheritance matrix serves as input for the ‘Traveling Salesman Algorithm’ implemented in R package TSP (version 1.1–7)^41^ and attempts to order contigs based on strand state coinheritance. As the initial squashed assembly might contain assembly errors, SaaRclust is able to detect and correct such errors as bins of the same contig being assigned to different clusters (‘Chimeric contig’) or bins of the same contig that differ in directionality (‘Misoriented contig’). Lastly, we export clustered, reoriented and ordered contigs into a single FASTA file with a single FASTA record per cluster. A complete list of parameters used to run SaaRclust in this study is reported below:

SaaRclust command:

scaffoldDenovoAssembly(bamfolder = <>, outputfolder = <>, store.data.obj = TRUE, reuse.data.obj = TRUE, pairedEndReads = TRUE, bin.size = 200000, step.size = 200000, prob.th = 0.25, bin.method = ’dynamic’, min.contig.size = 100000, assembly.fasta = assembly.fasta, concat.fasta = TRUE, num.clusters = 100|150, remove.always.WC = TRUE, mask.regions = FALSE)

### Variant calling

Clustered assemblies in full chromosomal scaffolds are then used for realignment of long PacBio reads. To call variants in HiFi reads, we use DeepVariant^38^ v0.9.0, which uses a deep neural network with a pre-trained model (--model_type=PACBIO). For the variant calling, HiFi reads were aligned using pbmm2 v1.1.0 (https://github.com/PacificBiosciences/pbmm2) with settings align --log-level DEBUG --preset CCS --min-length 5000 and filtered with samtools view -F 2308. After variant calling, we select only heterozygous SNVs using BCFtools v1.9.

For both PacBio CLR and ONT reads, we use the LongShot variant caller:

longshot --no_haps --force_overwrite --auto_max_cov--bam {alignments} --ref {clustered_assm}--region {contig} --sample_id {individual} --out {output}

### Phasing chromosomal scaffolds

To create completely phased chromosomal scaffolds, we used a combination of Strand-seq and long-read phasing^18^. First, we realigned Strand-seq data on top of the clustered assemblies as stated previously. Only regions that inherit a Watson and Crick template strand from each parent are informative for phasing and are detected using breakpointR^42^. Haplotype-informative regions are then exported using the breakpointR function called ‘exportRegions’. Using the set of haplotype-informative regions together with positions of heterozygous SNVs, we ran StrandPhaseR^18^ to phase SNVs into whole-chromosome haplotypes. Such sparse haplotypes are then used as a haplotype backbone for long-read phasing using WhatsHap to increase density of phased SNVs.

breakpointR command (run and export of results):

breakpointr(inputfolder = <>, outputfolder = <>, windowsize = 500000, binMethod = ’size’, pairedEndReads = TRUE, pair2frgm = FALSE, min.mapq = 10, filtAlt = TRUE, background = 0.1, minReads = 50)exportRegions(datapath = <>, file = <>, collapseInversions = TRUE, collapseRegionSize = 5000000, minRegionSize = 5000000, state = ’wc’)

StrandPhaseR command:

strandPhaseR(inputfolder = <>, positions = <SNVs.vcf>, WCregions = <hap.informtive.regions>, pairedEndReads = TRUE, min.mapq = 10, min.baseq = 20, num.iterations = 2, translateBases = TRUE, splitPhasedReads = TRUE)

WhatsHap command:

whatshap phase --chromosome {chromosome} --reference {reference.fasta} {input.vcf} {input.bam} {input.vcf_sparse_haplotypes}

### Haplotagging PacBio reads

Having completely phased chromosomal scaffolds at sufficient SNV density allows us to split long PacBio reads into their respective haplotypes using WhatsHap. This step can be performed in two ways: splitting all reads across all clusters into two bins per haplotype or splitting reads into two bins per cluster and per haplotype. Both strategies consist of the same two steps: 1) labeling all reads with their respective haplotype (‘haplotagging’) and 2) splitting the input reads only by haplotype or by haplotype and cluster (‘haplosplitting’). The WhatsHap commands are identical in both cases except for limiting WhatsHap to a specific cluster during haplotagging and discarding reads from other clusters to separate the reads by haplotype and cluster:

whatshap haplotag [--regions {cluster}] --output {output.bam} --reference {input.fasta} --output-haplotag-list {output.tags}{input.vcf} {input.bam}whatshap split [--discard-unknown-reads] --pigz --output-h1 {output.hap1} --output-h2 {output.hap2} --output-untagged {output.un} --read-lengths-histogram {output.hist} {input.fastq} {input.tags}

### Creating haplotype-specific assemblies

After haplotagging and haplosplitting, the long HiFi reads separated by haplotype were then used to create fully haplotype-resolved assemblies. Our haplotagging and haplosplitting strategy enabled us to examine two types of haploid assemblies per input long-read dataset: the two haplotype-only assemblies (short: h1 and h2), plus the haploid assemblies created by using also all untagged reads—that is, all reads that could not be assigned to a haplotype (short: h1-un and h2-un). Hence, for each input read dataset, this amounts to four ‘genome-scale’ assemblies. We focused our analyses on the read sets h1-un (H1) and h2-un (H2). Final phased assemblies were created using parameters stated in the ‘Squashed genome assembly’ section.

### SD analysis

SDs were defined as resolved or unresolved based on their alignments to SDs defined in GRCh38 (http://genome.ucsc.edu/cgi-bin/hgTables?db=hg38&hgta_group=rep&hgta_track=genomicSuperDups&hgta_table=genomicSuperDups&hgta_doSchema=describe+table+schema) using minimap2 with the following parameters: --secondary=no -a --eqx -Y -x asm20 -m 10000 -z 10000,50 -r 50000 --end-bonus=100 -O 5,56 -E 4,1 -B 5 (ref. ^33^). Alignments that extended a minimum number of base pairs beyond the annotated SDs were considered to be resolved. The percent of resolved SDs was determined for minimum extension varying from 10,000 to 50,000 bp, and the average was reported. This analysis is adapted from Vollger et al.^34^ (https://github.com/mrvollger/segdupplots).

### SD collapse analysis

Collapses were identified using the methods described in Vollger et al.^34^. In brief, the method identifies regions in the assemblies that are at least 15 kbp in length and have read coverage exceeding the mean coverage plus three standard deviations. Additionally, collapses with more than 75% common repeat elements (identified with RepeatMasker) or TRs (identified with Tandem Repeats Finder^43^) are excluded.

### BAC clone insert sequencing

BAC clones from the VMRC62 clone library were selected from random regions of the genome not intersecting with an SD (*n* = 77). DNA from positive clones were isolated, screened for genome location and prepared for long-insert PacBio sequencing as previously described (Segmental Duplication Assembler (SDA))^34^. Libraries were sequenced on the PacBio RS II with P6-C4 chemistry (17 clones) or the PacBio Sequel II with Sequel II 2.0 chemistry (S/P4.1-C2/5.0-8 M; 60 clones). We performed de novo assembly of pooled BAC inserts using Canu v1.5 (Koren et al.^26^) for the 17 PacBio RS II BACs and using the PacBio SMRT Link v8.0 Microbial assembly pipeline (Falcon + Raptor, https://www.pacb.com/support/software-downloads/) for the 60 Sequel II BACs. After assembly, we removed vector sequence pCCBAC1, re-stitched the insert and then polished with Quiver or Arrow. Canu is specifically designed for assembly with long error-prone reads, whereas Quiver/Arrow is a multi-read consensus algorithm that uses the raw pulse and base-call information generated during SMRT (single-molecule, real-time) sequencing for error correction. We reviewed PacBio assemblies for misassembly by visualizing the read depth of PacBio reads in Parasight (http://eichlerlab.gs.washington.edu/jeff/parasight/index.html), using coverage summaries generated during the resequencing protocol.

### Assembly polishing and error correction

Assembly misjoints are visible using Strand-seq as recurrent changes in strand state inheritance along a single contig. Strand state changes can result from a double-strand break (DSB) repaired by homologous recombination during DNA replication, causing an SCE^1^. DSBs are random independent events that occur naturally during a cell’s lifespan and, therefore, are unlikely to occur at the same position in multiple single cells^2^. Instead, a strand state change at the same genomic position in a population of cells is indicative of a different process other than DSB (such as a genomic SV or genome misassembly)^13,44,45^. Observing a complete switch from WW (Watson–Watson) to CC (Crick–Crick) strand state or vice versa at about 50% frequency is observed when a part of the contig is being misoriented (Supplementary Fig. 6). All detected misassemblies in the final phased assemblies (Supplementary Table 1) were corrected using SaaRclust using the following parameters:

scaffoldDenovoAssembly(bamfolder = <>, outputfolder = <>, store.data.obj = TRUE, reuse.data.obj = TRUE, pairedEndReads = TRUE, bin.size = 200000, step.size = 200000, prob.th = 0.9, bin.method = ’dynamic’, ord.method = ’greedy’, min.contig.size = 100000, min.region.to.order = 500000, assembly.fasta = assembly.fasta, concat.fasta = FALSE, num.clusters = 100|150, remove.always.WC = TRUE, mask.regions = FALSE)

### Common assembly breaks

To detect recurrent breaks in our assemblies, we searched for assembly gaps present in at least one phased assembly completed by Flye (for CLR PacBio reads) or Peregrine (for HiFi PacBio reads). For this, we mapped all haplotype-specific contigs to GRCh38 using minimap2 using the same parameters as in the SD analysis method. We defined an assembly break as a gap between two subsequent contigs. We searched for reoccurring assembly breaks in 500-kbp non-overlapping bins and filtered out contigs smaller than 100 kbp. Each assembly break was defined as a range between the first and the last breakpoint found in any given genomic bin and was annotated based on the overlap with known SDs, gaps, centromeres and SV callsets^19^, allowing overlaps within 10-kbp distance from the breakpoint boundaries.

### Base accuracy

Phred-like QV calculations were made by aligning the final assemblies to 77 sequenced and assembled BACs from VMRC62 falling within unique regions of the genome (>10 kbp away from the closest SD) where at least 95% of the BAC sequence was aligned. The following formula was used to calculate the QV, and insertions and deletions of size *N* were counted as *N* errors: QV = –10log_10_(1 – (percent identity/100)).

Each assembly was polished twice with Racon^28^ using the haplotype-partitioned HiFi FASTQs. The alignment and polishing steps were run with the following commands:

minimap2 -ax map-pb --eqx -m 5000 -t {threads} --secondary=no {ref} {fastq} | samtools view -F 1796 - > {sam}racon {fastq} {sam} {ref} -u -t {threads} > {output.fasta}

The HG00733 ONT assemblies were polished with MarginPolish/HELEN^32^ (git commit 4a18ade) following developer recommendations. The alignments were created with minimap2 v2.17 and used for polishing as follows:

minimap2 -ax map-ont -t {threads} {assembly} {reads} | samtools sort -@ {threads} |samtools view -hb -F 0×104>{output}marginpolish {alignments} {assembly} MP_r941_guppy344_human.json--threads {threads} --produceFeatures --outputBase {output}helen polish --image_dir {mp_out} --model_path HELEN_r941_guppy344_human.pkl--threads {threads} --output_dir {output} --output_prefix HELEN

QV estimates based on variant callsets lifted back to the human reference hg38 were derived as follows: Genome in a Bottle^46^ high-confidence region sets (release v3.3.2) for individuals HG001, HG002 and HG005 were downloaded, and the intersection of all regions (BEDTools v2.29.0 ‘multiinter’^47^) was used as proxy for high-confidence regions in other samples (covering ~2.17 Gbp). For all samples, variant callsets based on Illumina short-read alignments against the respective haploid assembly were generated using BWA 0.7.17 and FreeBayes v1.3.1 as follows:

bwa mem -t {threads} -R {read_group} {index_prefix} {reads_mate1} {reads_mate2} | samtools view -u -F 3840 - |samtools sort -l 6 {output_bam}

The BAM files were sorted with SAMtools v1.9 and duplicates marked with Sambamba v0.6.6 ‘markdup’. The variant calls with FreeBayes were generated as follows:

freebayes --use-best-n-alleles 4 --skip-coverage {cov_limit} --region {assembly_contig} -f {assembly_fasta}--bam {bam_child} --bam {bam_parent1} --bam {bam_parent2}

Options ‘--use-best-n-alleles’ and ‘--skip-coverage’ were set following developer recommendations to increase processing speed. Variants were further filtered with BCFtools v1.9 for quality and read depth: ‘QUAL >=10 && INFO/DP<MEAN+3*STDDEV’. Variants were converted into BED format using vcf2bed v2.4.37 (ref. ^48^) with parameters ‘--snvs’, ‘--insertions’ and ‘--deletions’. The alignment information for lifting variants from the haploid assemblies to the human hg38 reference was generated with minimap v2.17-r941, and the liftover was realized with paftools (part of the minimap package):

minimap2 -t {threads} -cx asm20 --cs --secondary=no -Y -m 10000 -z 10000,50 -r 50000 --end-bonus=100 -O 5,56 -E 4,1 -B 5 ’ hg38.fasta {input_hap_assembly} > {hap-assm}_to_hg38.pafpaftools.js liftover -1 10000 {input_paf} {input_bed} > {output.hg38.bed}

The lifted variants were intersected with our custom set of high-confidence regions using BEDTools ‘intersect’. The total number of base pairs in homozygous variants was then computed as the sum over the length (as reported by FreeBayes as LEN) of all variants located in the high-confidence regions. Because not all variants could be lifted from the haploid to the hg38 reference assembly, we cannot know whether these variants would fall into the ‘high-confidence’ category. We thus computed a second, more conservative, QV estimate counting also all homozygous calls as error that were not lifted to the hg38 reference.

### Hi-C based scaffolding and validation

To independently evaluate the accuracy of our scaffolds, we used proximity ligation data for NA12878 and HG00733 (‘Data availability’). By aligning Hi-C data to our scaffolds produced by SaaRclust, we can visually confirm that long-range Hi-C interactions are limited to each cluster reported by SaaRclust.

In addition, we attempted to reproduce Hi-C-based scaffolds presented by Garg et al.^12^ for NA12878 using 3D-DNA^49^. Input to this pipeline was created with Juicer^50^ and an Arima Genomics Hi-C script, which are both publicly available.

Arima script

generate_site_positions_Arima.py -i {squashed_asm} -e {cut-Sequence} -o {cut-sites.txt}

Juicer

juicer.sh -g {genome_id} -s {enzyme} -z {squashed_asm} -r -p {chrom.sizes} -y {cut-sites.txt}

3D-DNA

run-asm-pipeline.sh {squashed_asm} {juicer_merged_no_dups}

### SV, indel and SNV detection

Methods for SV, indel and SNV calling are similar to previous HiFi assembly work^33^ but were adapted for phased assemblies. Variants were called against the GRCh38 primary assembly (that is, no alternate, patch or decoy sequences), which includes chromosomes and unplaced/unlocalized contigs. Mapping was performed with minimap2 2.17 (ref. ^51^) using parameters --secondary=no -a -t 20 --eqx -Y -x asm20 -m 10000 -z 10000,50 -r 50000 --end-bonus=100 -O 5,56 -E 4,1 -B 5, as described previously^33^. Alignments were then sorted with SAMtools v1.9 (ref. ^52^).

To obtain variant calls, alignments were processed with PrintGaps.py, which was derived in the SMRT-SV v2 pipeline (https://github.com/EichlerLab/smrtsv2)^53,54^, to parse CIGAR string operations to make variant calls^30^.

Alignment records from assemblies often overlap, which would produce duplicate variant calls with possible different representations (fragmented or shifted). For each haplotype, we constructed a tiling path covering GRCh38 once and traversing loci most centrally located within alignment records. Variants within the path were chosen, and variants outside the tiling path (that is, potential duplicates) were dropped from further analysis.

After obtaining a callset for H1 and H2 independently, we then merged the two haplotypes into a single callset. For homozygous SV and indel calls, an H2 variant must intersect an H1 variant by 1) 50% reciprocal overlap (RO) or 2) within 200 bp and a 50% reciprocal size overlap (RO if variants were shifted to maximally intersect). For homozygous SNV calls, the position and alternate base must match exactly. The result is a unified phased callset containing homozygous and heterozygous variants. Finally, we filtered out variants in pericentromeric loci where callsets are difficult to reproduce^54^ and loci where we found a collapse in the assembly of either haplotype.

We intersected RefSeq annotations from the UCSC RefSeq track and evaluated the effect on genes noting frameshift SVs and indels in coding regions by quantifying the number of bases affected per variant on genic regions. If an insertion or deletion changes coding sequence for any isoform of a gene by a non-modulo-3 number of bases, we flag the gene as likely disrupted.

Variants falling within TRs and SDs were also annotated using UCSC hg38 tracks. For TR and SD BED files, we merged records allowing regions within 200 bp to overlap with BEDTools^47^. SVs and indels that were at least 50% contained within an SD or TR region were annotated as SD or TR. For RefSeq analysis, we classified genes as contained within TR or SD by intersecting exons with the collapsed TR and SD regions allowing any overlap.

### Phasing accuracy estimates

To evaluate phasing accuracy, we determined SNVs in our phased assemblies based on their alignments to GRCh38. This procedure is described in the ‘SV, indel and SNV detection’ section in the Methods. We evaluate phasing accuracy of our assemblies in comparison to trio-based phasing for HG00733 (ref. ^19^) and NA12878 (ref. ^46^). In all calculations, we compare only SNV positions that are shared between our SNV calls and those from trio-based phasing. To count the number of switch errors between our phased assemblies and trio-based phasing, we compare all neighboring pairs of SNVs along each haplotype and recode them into a string of 0s and 1s depending on whether the neighboring alleles are the same (0) or not (1). The absolute number of differences in such binary strings is counted between our haplotypes and the trio-based haplotypes (per chromosome). The switch error rate is reported as a fraction of counted differences of the total number of compared SNVs (per haplotype). Similarly, we calculate the Hamming distance as the absolute number of differences between our SNVs and trio-based phasing (per chromosome) and report it as a fraction of the total number of differences to the total number of compared SNVs (per haplotype).

### MHC analysis

We extracted the MHC, defined as chr6:28000000–34000000, by mapping each haplotype sequence against GRCh38 and extracting any primary or supplementary alignments to this region. We created a dotplot for each haplotype’s MHC region using Dot from DNAnexus (https://github.com/dnanexus/dot) (Supplementary Fig. 11). We created phased VCFs for both the CCS and Shasta assemblies using the two haplotype files as input to Dipcall (https://github.com/lh3/dipcall). Then, we compared the phasing between the haplotype files using the compare module within WhatsHap. This results in a switch error rate of 0.48% (six sites) and a Hamming error rate of 0.28% (four sites) from 1,433 common heterozygous sites between the VCFs.

### Detection of loss of heterozygosity regions

To localize regions of decreased heterozygosity, we calculated the SNV diversity as a fraction of heterozygous variants between H1 and H2 within 200-kbp-long genomic bins (sliding by 10 kbp). In the next step, we rescaled SNV diversity values to a vector of 0s and 1s by setting values <25th quantile to 0 and those >25th quantile to 1. Then, we used R package fastseg^55^ to find change points in previously created vector of 0s and 1s while reporting segments of minimal length of 200 (diversity values per bins). In turn, we genotyped each segment based on a median segment value. Segments with median value ≤0.05 were genotyped as ‘LOH’ (loss of heterozygosity), whereas the rest were genotyped as ‘NORM’ (normal level of heterozygosity).

### Detection of misassembled contigs

To detect assembly errors in squashed or phased assemblies, we used our SaaRclust package. First, we aligned Strand-seq reads to an assembly in question and then ran SaaRclust with the following parameters:

scaffoldDenovoAssembly(bamfolder = <>, outputfolder = <>, store.data.obj = TRUE, reuse.data.obj = TRUE, pairedEndReads = TRUE, bin.size = 200000, step.size = 200000, prob.th=0.25, bin.method = ’fixed’, ord.method = ’greedy’, min.contig.size = 100000, assembly.fasta = assembly.fasta, concat.fasta = FALSE, num.clusters = 100, remove.always.WC = TRUE, mask.regions = FALSE, desired.num.clusters = 24)

The list of misassembled contigs predicted assembly errors is reported by SaaRclust in RData object with prefix ‘putativeAsmErrors_*’.

### Likely disrupted genes

Using RefSeq intersect counts, we found all genes with at least one non-modulo-3 insertion or deletion within the coding region of any isoform (that is, frameshift). We filtered out any genes not fully contained within a consensus region of the two haplotypes, which we defined as regions where both H1 and H2 had exactly one aligned contig. If a gene had multiple non-modulo-3 events, whether in the same isoform or not, the gene was counted once.

### Variant comparisons

We compared variants to previously published callsets by intersecting them with the same RO/Size-RO strategy used to merge haplotypes. For HGSVC comparisons, we also excluded variant calls on unplaced contigs, unlocalized contigs and chrY of the reference (that is, chr1-22,X), which were not reported by the HGSVC study. To quantify the number of missed variants proximal to another, we took variants that failed to intersect an HGSVC variant and found the distance to the nearest variant of the same type (INS versus INS and DEL versus DEL).

### Robust and reproducible implementation

The basic workflow of our study is implemented in a reproducible and scalable Snakemake^56^ pipeline that has been successfully tested in compute environments ranging from single servers to high-performance cluster setups (‘Code availability’). Major tasks in the pipeline, such as read alignment or assembly, have been designed as self-contained ‘start-to-finish’ jobs, automating even trivial steps, such as downloading the publicly available datasets used in this study. Owing to the considerable size of the input data, we strongly recommend deploying this pipeline only on compute infrastructure tailored to resource-intensive and highly parallel workloads.

### Reporting Summary

Further information on research design is available in the Nature Research Reporting Summary linked to this article.

## Online content

Any methods, additional references, Nature Research reporting summaries, source data, extended data, supplementary information, acknowledgements, peer review information; details of author contributions and competing interests; and statements of data and code availability are available at 10.1038/s41587-020-0719-5.

## Supplementary information

Supplementary Information Supplementary Notes, Supplementary Figs. 1–24, Supplementary Tables 2–5 and 7 and HGSV Consortium Members.

Reporting Summary

Supplementary Table 1 De novo assembly statistics. Columns B–F, basic characteristics for each phased assembly (column A). Column F, the contig N50 value is taken from QUAST-LG analysis reports. Columns G,H, switch error and Hamming distance computed as described in the Methods. Columns J,K, Illumina-based QV estimates (Methods) counting only HOM SNV (column J) or all HOM variant calls (column K) as errors in the phased assembly. The right QV estimate is computed based only on variants in high-confidence regions (Methods); the left number additionally takes variant calls into account that were not lifted to the GRCh38 reference. Column L, parameter set used to generate the respective assembly; see the pipeline repository (Code availability). Column M, FASTA file name of phased assembly (Data availability).

Supplementary Table 6 List of detected UABs.

Supplementary Table 8 Accession IDs for data used in this study.

## Data Availability

HiFi PacBio reads for HG00731, HG00732 and HG00733 were produced as part of this study. A complete list of new and publicly available data used in this study is summarized in Supplementary Table 8. All phased assemblies listed in Supplementary Table 1 are available via the IGSR FTP at ftp.1000genomes.ebi.ac.uk/vol1/ftp/data_collections/HGSVC2/working/20200417_Marschall-Eichler_NBT_hap-assm/.

## Code availability

R package SaaRclust (MIT License): https://github.com/daewoooo/SaaRclust (devel branch); R package breakpointR (MIT License): https://bioconductor.org/packages/breakpointR/; R package StrandPhaseR (MIT License): https://github.com/daewoooo/StrandPhaseR (devel branch); Snakemake pipeline (MIT License): https://github.com/ptrebert/project-diploid-assembly (development branch); Custom R functions (MIT License): https://github.com/daewoooo/DiploidAssembly_paper; Assembly graph analysis (MIT License): https://github.com/natir/project-diploid-assembly-UAB-graph-analysis.
