# Supplementary material for: Fully phased human genome assembly without parental data using single-cell strand sequencing and long reads
Source: Nat Biotechnol. 2020 Dec 7;39(3):302–8. doi: 10.1038/s41587-020-0719-5 (PMC7954704; doi:10.1038/s41587-020-0719-5)
Supplement: Supplementary file 1 — Supplementary Notes, Supplementary Figs. 1–24, Supplementary Tables 2–5 and 7 and HGSV Consortium Members. [file 41587_2020_719_MOESM1_ESM.pdf]

natureresearch

https://doi.org/10.1038/s41587-020-0719-5

Supplementary information

Fully phased human genome assembly without parental data using single-cell strand sequencing and long reads

In the format provided by the authors and unedited

Supplementary materials

Fully phased human genome assembly without parental data using single-cell Strand-seq and long reads

Porubsky, Ebert et al.

Supplementary Notes

Strand-seq

The Strand-seq protocol utilizes a thymidine analog to selectively label and remove one of the DNA strands (the nascent strand, synthesized during DNA replication) and leaves only the template DNA strands intact for sequencing. By sequencing only template strands in each homologue, Strand-seq distinguishes three possible template strand states for each chromosome of a diploid genome. The Watson-Watson (WW) strand state is characteristic of two Watson (reads aligned to minus strand) templates inherited from both parental homologues. The Crick-Crick (CC) strand state is characteristic of two Crick (reads aligned to plus strand) templates inherited from both parental homologues. Lastly, the Watson-Crick (WC) strand state is characteristic of a Watson and Crick template being inherited from either parental homologue. In this WC scenario the two parental templates can be distinguished based on read directionality and are thus informative of phasing. Template strands are randomly inherited by single daughter cells, resulting in a specific strand-state pattern for each chromosome across multiple Strand-seq libraries (**Fig. 1b**). This strand-state pattern can be viewed as a barcode that uniquely assigns each contig to its chromosome of origin (Hills et al. 2013) (**Fig. 1c**). However, we do not always observe a single strand state along the whole chromosome but instead there can be strand-state changes as a result of a double-strand break (DSB) repaired by sister chromatid exchange (SCE) during DNA replication (Falconer et al. 2012; van Wietmarschen and Lansdorp 2016; Claussin et al. 2017). Such low-frequency SCEs are indicative of the physical distance between two segments of a chromosome, because segments that are physically further apart from each other have an increased likelihood of an SCE occurring between them (Hills et al. 2013). This means that contigs that are physically linked to each other are less likely to be separated by SCEs and, thus, will share the same strand state across multiple cells—a signal that enables assembled contigs to be clustered into chromosomes and then ordered within each chromosome (**Fig. 1d**). Clustered and ordered contigs can then be phased using single-nucleotide polymorphism information extracted from the haplotype-informative (WC) regions in the Strand-seq data (**Fig. 1e**). This allows us to physically separate parental alleles along the whole chromosome (Porubský et al. 2016). Such global phasing information in conjunction with long-read technologies such as PacBio allows us to reconstruct highly accurate and nearly complete haplotypes that span the whole chromosomes (Porubsky et al. 2017; Chaisson et al. 2019). Such haplotypes serve as a guide to divide long-read data into two bins, one for each haplotype (**Fig. 1f**).

SaaRclust

Every chromosome undergoes independent random segregation during cell division, leading to a unique strand-state profile in Strand-seq data. This signal in Strand-seq data can be employed to cluster long sequencing reads by chromosome of origin and sequencing direction. SaaRclust (Ghareghani et al. 2018) is a tool we previously introduced for this *in silico* separation of long reads by chromosome and direction. SaaRclust employs an expectation-maximization (EM) soft clustering algorithm to handle the uncertainty arising from the sparse Strand-seq data. Given the central importance of SaaRclust for the assembly pipeline we introduce here, we include Supplementary Figure 24 illustrating the principle. The main idea underlying our clustering algorithm is that contigs originating from the same chromosome (Contigs 1 and 2 in **Supplementary Fig. 24**) show the same directionality pattern of aligned Strand-seq reads across single cells, which is different for contigs originating from different chromosomes (contig 3 in **Supplementary Fig. 24**). The EM algorithm is based on iterating between assigning strand states for each Strand-seq library and chromosome and assigning chromosomes to each contig, which are both hidden information at the beginning. EM converges to a local optimum solution of the maximum likelihood problem, e.g., maximizing the likelihood of observed data (number of directional aligned Strand-seq reads to long reads), given the model parameters (strand states), and we have shown SaaRclust to be able to assign even individual long reads to chromosomes of origin. Here, we have adjusted it to work on the contig level. SaaRclust assigns each contig to a separate cluster defined by a unique strand inheritance over multiple Strand-seq libraries (**Fig. 1c**). Ideally each cluster represents a single chromosome, however, this notion is not always true for very small clusters, such as cluster 13 and 22 in Supplementary Fig. 2b. These clusters likely contain short contigs from repetitive regions of the genome that are difficult to assign to a single unique cluster.

Runtime and hardware requirements

Runtime and hardware requirements vary considerably depending on the type of the long reads used as input (e.g., PacBio HiFi/CCS or Oxford Nanopore [ONT]). Irrespective of the type of the reads, generating the initial unphased (“squashed”) assembly requires the single largest amount of resources. The ~34-fold coverage HG00733 HiFi/CCS data, for example, were assembled with Peregrine using 36 cores (~368 CPU hours) and ~600 GB RAM; the ~80x HG00733 ONT reads were assembled with Flye using 48 cores (~3700 CPU hours) and ~850 GB RAM. From other experiments with PacBio CLR reads, we know that similar requirements need to be met for assembling ~90x CLR datasets with Flye. For a complete pipeline run using ~30x HiFi/CCS reads as input, the total runtime is estimated at 2000 CPU hours. We note, however, that this estimate ignores the time required for preprocessing steps, such as downloading input and reference data, and also neglects evaluation steps such as QUAST analysis runs for all generated assemblies. Since we tested our pipeline on different compute infrastructures, we also know that there is a substantial I/O burden generated by some tools, e.g., by the Peregrine assembler. Based on our own experience, for slow (often network) file systems, this can easily double the overall runtime because certain steps in the pipeline spend most of the time performing I/O operations.

Variant discovery and comparisons

The single-nucleotide variant (SNV) transition/transversion (Ti/Tv) proportion was 1.99, 1.98, and 1.98 for h1, h2, and merged callsets, respectively. Outside of tandem repeats, the Ti/Tv rose to 2.05.

For variants that did not intersect an HGSVC call, we find that 78% (3,079 of 3,945) of false insertions and 75% (1,527 of 2,048) of false deletions map within 1 kbp of a variant of the same type indicating that many of these calls may be different representations of the same event but signify inconsistent alignment as discussed previously. Squashed assemblies, even when reference-guided, miss a large proportion heterozygous SV calls (Huddleston et al. 2017), and compared to a haplotype-unaware analysis of HG00733 (Audano et al. 2019), we find 31% and 12% more insertions and deletions outside repetitive loci, respectively.

Availability of Strand-seq to scientific community

Having Strand-seq data available for the test genome is central to this assembly approach. The generation of Strand-seq data has become practical with the introduction of automation procedures that utilize widely available hardware. A complete description of the steps required for automation are fully outlined by Sanders et al. in *Nature Protocols* (Sanders et al. 2017). Data production facilities, where Strand-seq library preparation has already been automated on robotic platforms, currently exist in Vancouver, Canada, Groningen, the Netherlands, and Heidelberg, Germany. Upon implementation on a Biomek FX^P robotic liquid handler at the European Molecular Biology Laboratory (EMBL, Heidelberg), the protocol requires two days to process 96 barcoded single-cell libraries, with a combined reagent cost of ~$15 USD per cell. We are confident that with several recent publications showcasing important new application areas for this datatype, more centers will implement high-throughput versions of the Strand-seq protocol.

To apply this assembly method more broadly, it is important to note that while Strand-seq relies on dividing cells, its applicability indeed is not limited to cell lines. The Strand-seq protocol has already been adapted to various cancer and non-cancer/normal immortalized cell lines, primary leukemia-derived samples, xenografts, primary normal (skin) fibroblasts, and hematopoietic stem and progenitor cells (obtained from bone marrow or cord blood; some of these data are unpublished, others published (Porubský et al. 2016; Sanders et al. 2019, 2016)). Beyond these human materials, Strand-seq data by now have been generated for various nonhuman genomes, including a variety of different nonhuman primate samples (chimpanzee, bonobo, gorilla, orangutan, macaque and gibbon) (Porubsky et al. 2020), organoids, mouse, C. elegans, and yeast in the field. Because Strand-seq only requires one round of cell division, different clinical biopsy materials (including lymphoblasts from blood or skin cells) can be readily used, rendering assembly and phasing to work out samples of clinical interest feasible. We believe these data offer an exciting opportunity to apply our reference-free assembly methods to genomes of evolutionary, population-genetics or clinical interest.

Comparative assembly analysis

To compare the five diploid assemblies HG00731-HiFi, HG00732-HiFi, HG00733-HiFi, HG00733-CLR, and HG00733-ONT, we aligned the ten haplotype assemblies separately to GRCh38 using minimap2 and subsequently produced one variant callset for each haplotype assembly using paftools. We then restricted the callset to genomic regions where all ten assemblies had an alignment (in total 2.66 Gbp of sequence), merged the callsets across samples (turning overlapping variants into a multi-allelic representation in VCF) to produce a phased VCF with five samples, and normalized the VCF using bcftools. The code for creating callset is available from https://bitbucket.org/jana_ebler/vcf-merging.

The resulting callset had 10,697,583 variants in total. By comparing the three HG00733 assemblies, we found that the genotypes for 46.3% of all variants where concordant across all three. The discordancies where mostly caused by likely errors in the ONT assembly (sites where the genotypes of HiFi and CLR assemblies agree with one another but disagree with the ONT assembly), which amounted to 5,238,756 sites (48.97%). In contrast, we found only 106,270 (0.99%) such sites for CLR and 25,586 (0.24%) for HiFi, which is consistent with the relative order of QV estimates made for these assemblies (**Supplementary Table 1**). These results are further corroborated by observing that there are 5,601,071 (52.36%) Mendelian errors for ONT; 469,127 (4.39%) for CLR; and 131,281 (1.23%) for HiFi. Note that Mendelian errors can also be caused by assembly errors in the parents and the percentages are thus higher than for the concordance analysis above. Finally, we combined concordance and Mendelian consistency analysis and found that among all HiFi genotypes, there are only 82,616 (0.77%) that are neither supported by Mendelian consistency nor by any of the other two assemblies.

Overlap graph analysis

We follow the approach introduced by (Marijon, Chikhi, and Varré 2019b) to analyze problematic regions and selected a universal assembly break (UAB) at chr7:45,788,351-45,828,535 as an example: For each cluster, we map reads back to contigs and disregard those reads fully contained within contigs. For the remaining reads, we run an all-vs-all search for sequence overlaps both between reads and between reads and contigs using minimap2 (Li 2018). The set of overlaps is converted into an overlap graph (GFA1 format) with fpa (Marijon, Chikhi, and Varré 2019a) and an image of the subgraph around breakpoint is generated with Bandage (Wick et al. 2015) and shown in **Supplementary Figure 22c**. The code for this analysis is available at https://github.com/natir/project-diploid-assembly-UAB-graph-analysis.

Supplementary Figures 1-24

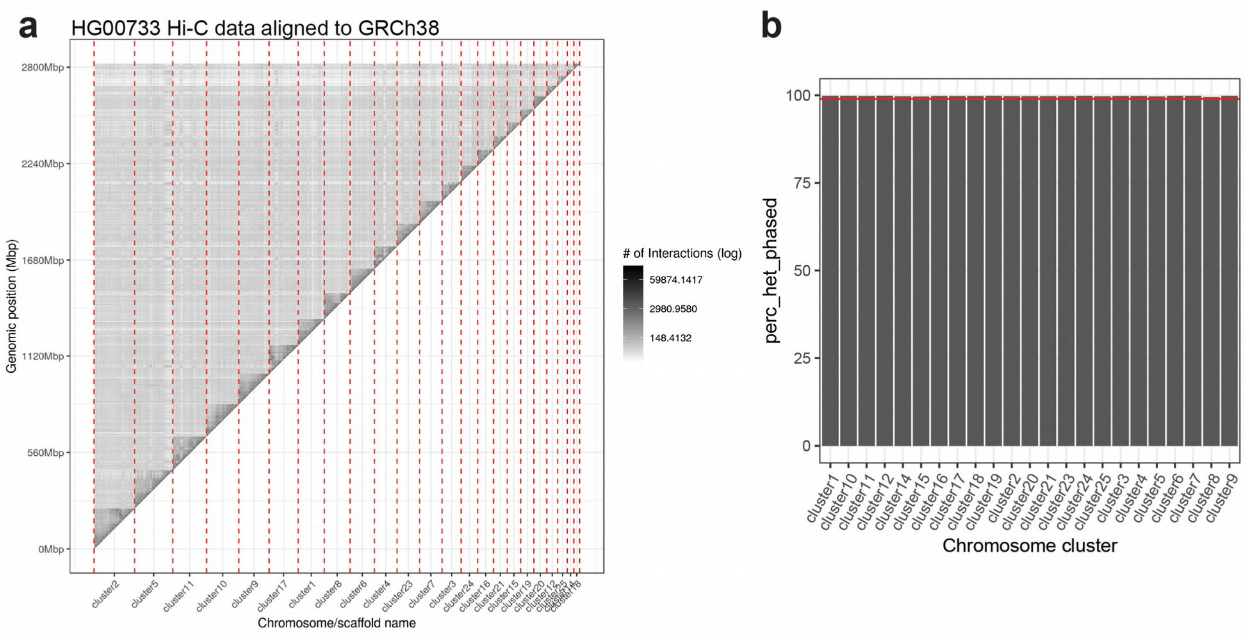

Supplementary Figure 1: Phasing of SNVs per chromosomal cluster.

a) A Hi-C contact matrix constructed from publicly available Hi-C data for HG00733 (**Data availability**) aligned to SaaRclust based chromosomal scaffolds made from squashed assemblies. **b**) Height of each bar represents the percentage of SNVs phased in the longest haplotype block in each cluster ('perc_het_phased'). Red line highlights the 99% threshold.

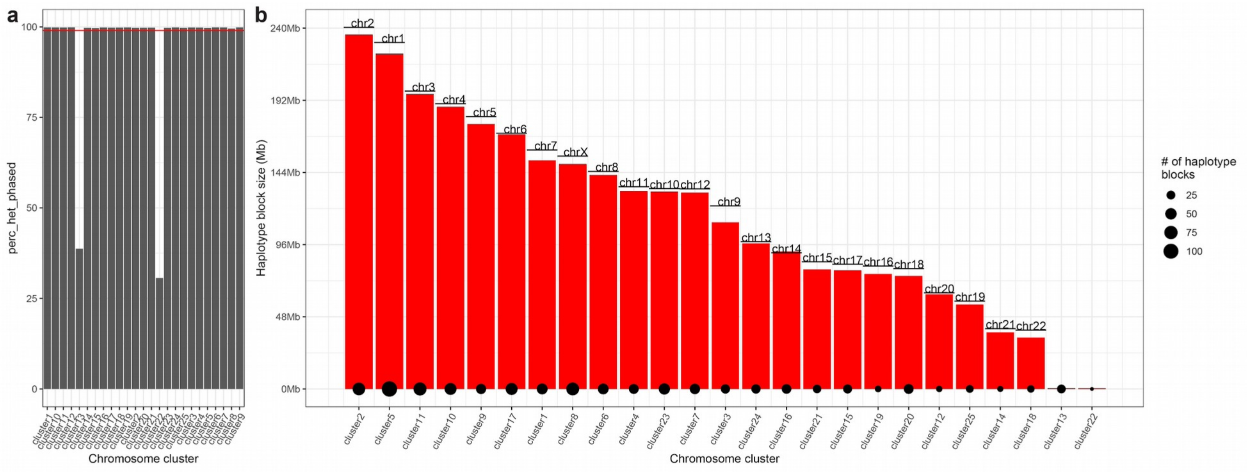

Supplementary Figure 2: Phasing of SNVs per chromosomal cluster (unfiltered data).

Phasing statistics for HG00733 before removing a very small clusters <1 Mbp of total length. Notice that there are two very small clusters (13 and 22)—as expected, these clusters have a very low number of phased heterozygous SNVs. These clusters contain contigs from repetitive regions where short Strand-seq reads do not map uniquely. **a**) Height of each bar represents the percentage of SNVs phased in the longest haplotype block in each cluster ('perc_het_phased'). The red line highlights the 99% threshold. **b**) A barplot that shows the total length of all haplotype blocks per cluster in dark gray. The dark gray bars are overlaid by the size of the longest haplotype block (shown in red) in each cluster. The size of the point at the bottom of each bar reflects the number of haplotype blocks in each cluster. For perspective, the real size of each chromosome for GRCh38 is plotted as a horizontal solid line.

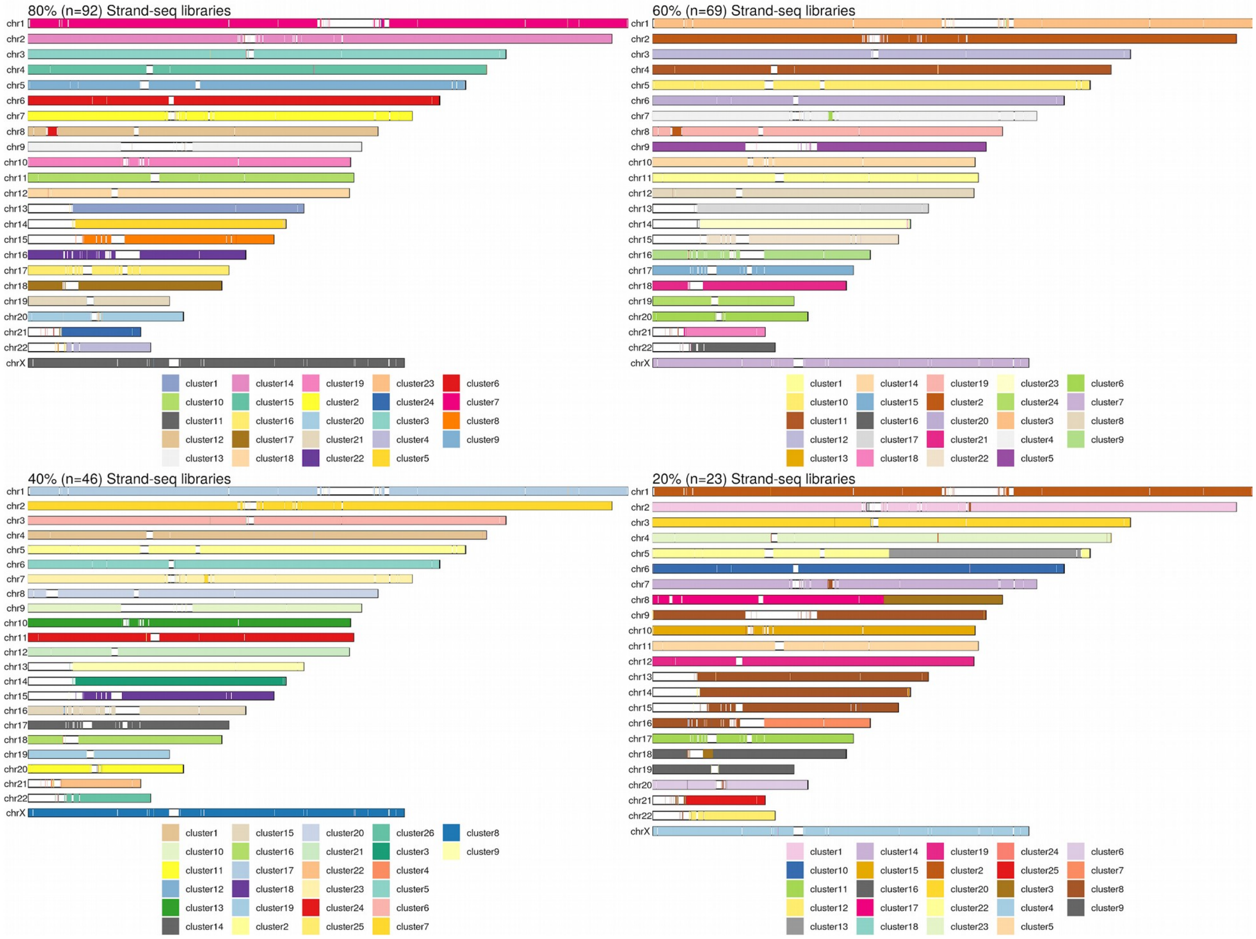

Supplementary Figure 3: Clustering accuracy using downsampled Strand-seq libraries. Here we have randomly selected 80%, 60%, 40% and 20% of the original number of Strand-seq libraries (n=115) and performed contig clustering using SaaRclust. Each contig represents a range based on mapping coordinates on GRCh38. Contigs are colored based on cluster identity determined by SaaRclust. In an ideal scenario there is a single color for each chromosome. When only 20% (n=23) of Strand-seq libraries were selected, we observe that we are no longer able to correctly assign contigs to a single chromosome.

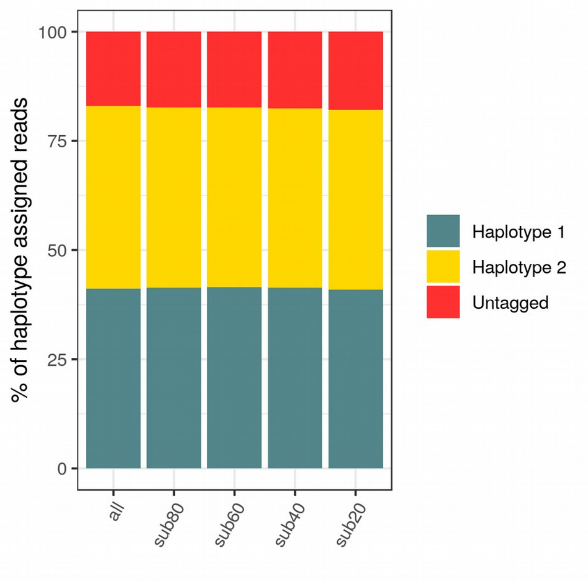

Supplementary Figure 4: Proportion of phased HiFi reads using downsampled Strand-seq libraries.

A barplot where each bar represents the stacked proportions of each read assigned to haplotype 1 and haplotype 2 and those could not be assigned (untagged) for different percentages (80, 60, 40 and 20) of the original number of Strand-seq libraries (n=115).

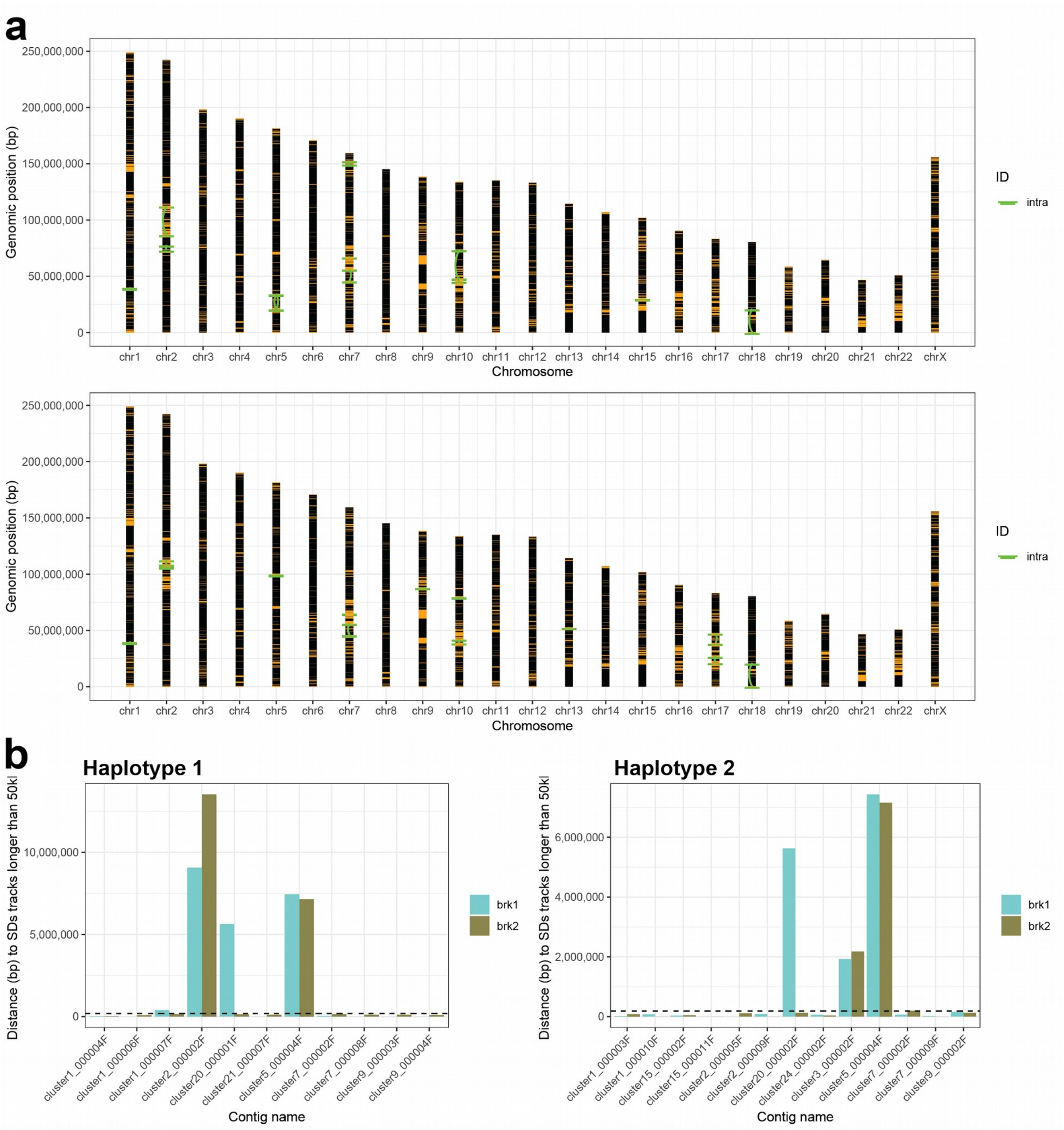

Supplementary Figure 5: Peregrine-specific assembly errors.

a) Projection of Peregrine-based assembly errors to GRCh38. Positions of segmental duplications (SDs) in the genome are highlighted in orange. Green links connect regions upstream and downstream from an assembly error (**Methods**). If no link is visible, the position upstream and downstream from the breakpoint lies in close proximity. NOTE: By performing phased assembly separately within each cluster, defined by SaaRclust, we avoid creation of chimeric contigs by Peregrine (only ‘intra’ errors). **b**) Each bar (turquoise - upstream from the assembly error, khaki - downstream from the assembly error) represents a distance to the closest SD track of 50 kbp and longer from the assembly error.

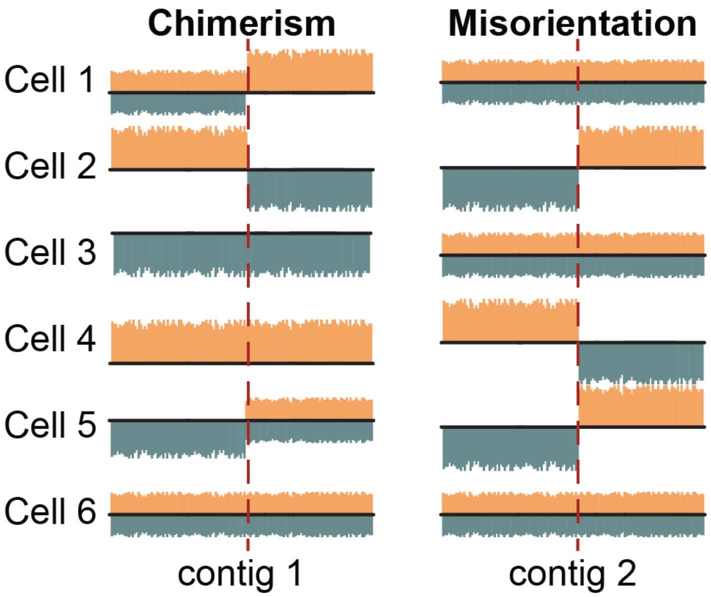

Supplementary Figure 6: Strand-seq patterns of common misassemblies.
A genome misassembly is visible in Strand-seq data as a recurrent change in strand state at the same position in a given contig. Because, it is highly unlikely for a DSB to occur at exactly the same position in multiple single cells, the most likely explanation in this case is either contig misorientation or chimerism. Chimerism is characteristic by almost all possible template strand changes (not just complete switch from WW to CC or vice versa). This is caused by the fact that portions of a chimeric contig that belong to different chromosome follow strand inheritance of a chromosome of origin. On the other hand, misorientation is characteristic of a complete switch from either WW to CC or vice versa. This type of misassembly is visible in about 50% of cells as only WW or CC template strand states are informative for this type of assembly error.

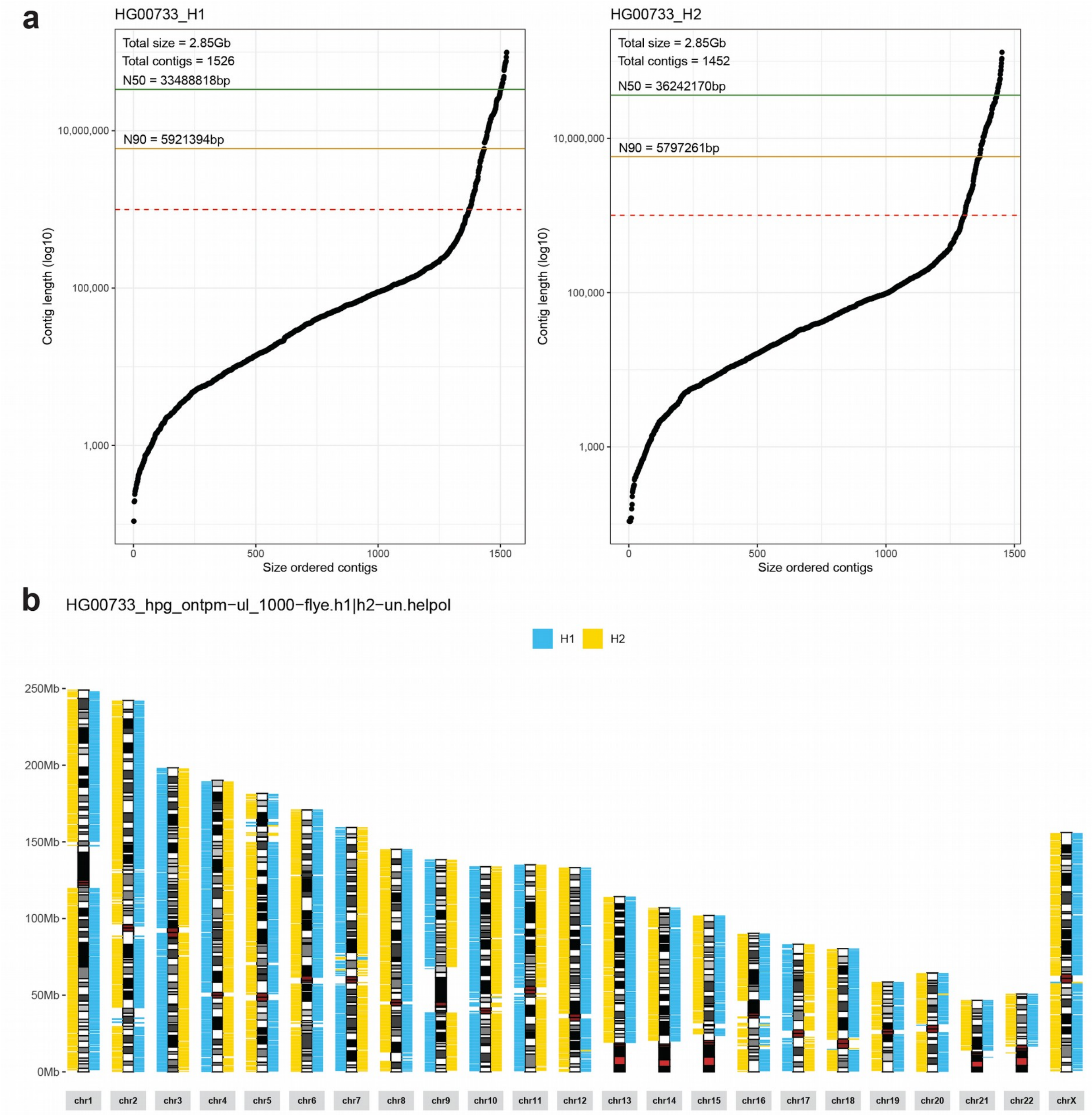

Supplementary Figure 7: Phased assembly of long error-prone ONT reads for HG00733.

a) Size distribution of haplotypes 1 and 2 specific contigs assembled using Flye. For both haplotypes, total assembly sizes, number of contigs, and assembly size N50s are reported. **b**) Each 1 Mbp block of phased contigs are assigned to one of the parental genomes using SNV data from the parents (Chaisson et al. 2019). HG00733 haplotypes are shown to the left (haplotype 1) and to the right (haplotype 2) from each chromosomal ideogram. Maternal segments (HG00732) are shown in blue and paternal segments (HG00731) are shown in yellow.

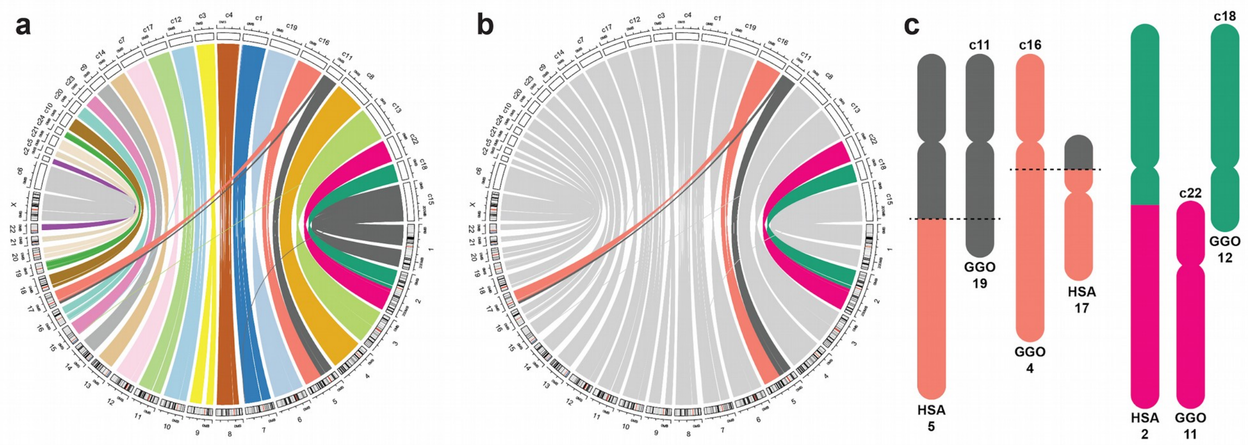

Supplementary Figure 8: Scaffolding gorilla genome using Strand-seq.
Circos plots comparing SaaRclust-based gorilla scaffolds (c1-c24) to GRCh38 (chromosomes 1-22 and X). GRCh38 chromosomes are shown as banded ideograms while gorilla scaffolds are shown in white colored ideograms. Comparison is made by nucmer alignment of gorilla scaffolds to GRCh38. **a**) Each line shows a nucmer alignment between gorilla scaffolds and GRCh38 and is uniquely colored by the gorilla scaffold it originates from. **b**) Highlighted reciprocal translocation between chromosomes 5 and 17 (in GRCh38) that are correctly scaffolded as 'c19' and 'c11' in gorilla. Another highlighted region is chromosome 2, which is as expected composed from two clusters ('c22' and 'c18') in the gorilla scaffold. **c**) A chromosomal model of a known reciprocal translocation between chromosomes 5 and 17 in human and precursor chromosomes that gave rise to chromosome 2 in humans (Jauch et al. 1992; Stankiewicz et al. 2004)**.**

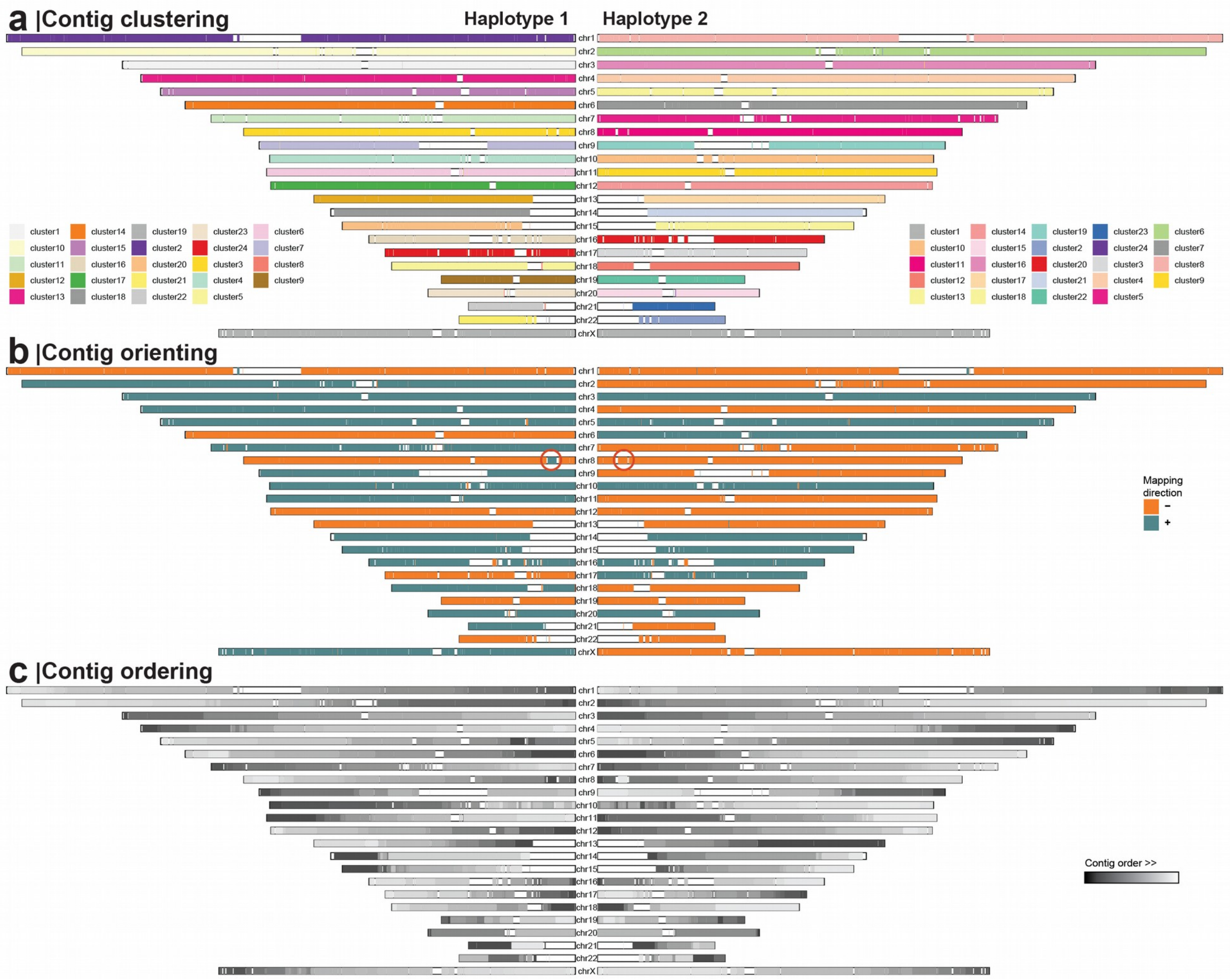

Supplementary Figure 9: Accuracy of contig clustering, orienting, and ordering per haplotype.

For this analysis we used only contigs 500 kbp and longer and those that can be assigned to a chromosomal cluster with probability >=0.9. **a**) Each contig represents a range based on mapping coordinates on GRCh38. Contigs are colored based on cluster identity determined by SaaRclust. In an ideal scenario there is a single color for each chromosome. **b**) Each contig represents a range based on mapping coordinates on GRCh38. Contigs are colored based on the directionality ('+' - positive strand, '-' - negative strand) they map to GRCh38. Ideally, there is a single color for each chromosome. Red circles indicate a known heterozygous inversion assigned here to haplotype 1. **c**) Each contig is colored based on the predicted order within each chromosomal cluster, which is reflected by the shades of gray going from dark to light gray. Ideally we observe colors going always from dark to light gray or vice versa and thus being in agreement with true contig order on GRCh38.

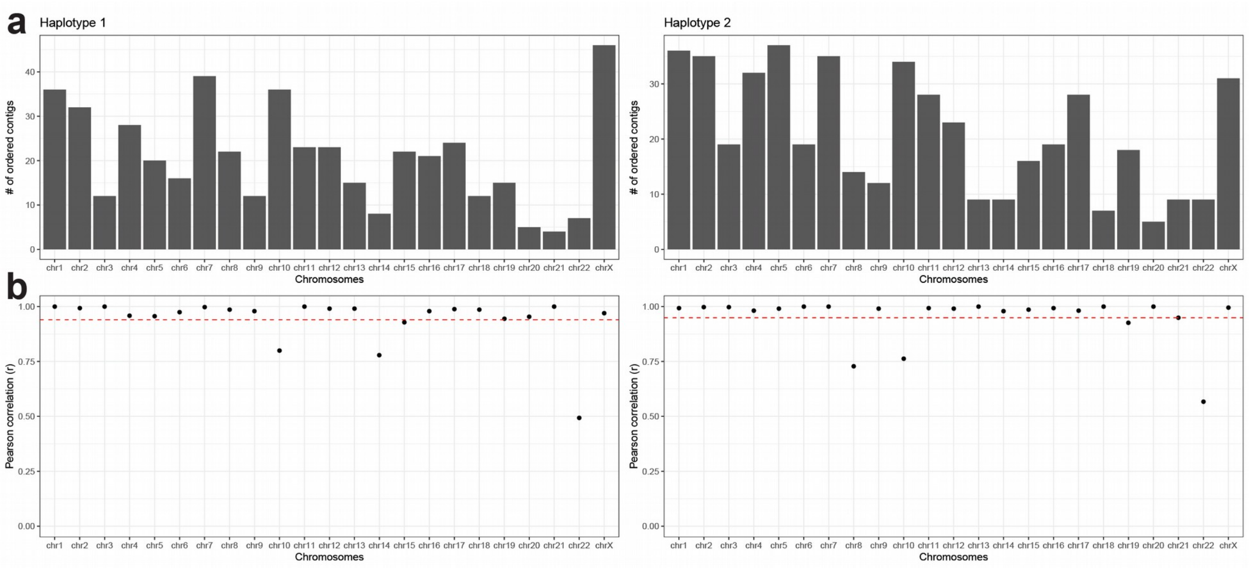

Supplementary Figure 10: Correlation of contig ordering with GRCh38.

a) Each bar represents a number of contigs submitted for ordering within each chromosome and haplotype. **b**) Correlation of predicted contig order with the expected ordering, based on GRCh38 mappings, within each chromosome and haplotype. Red dashed line shows mean correlation over all chromosome within a haplotype. NOTE: Contig order is reported as their relative distance only and do not always place start and end of each consecutive contigs correctly

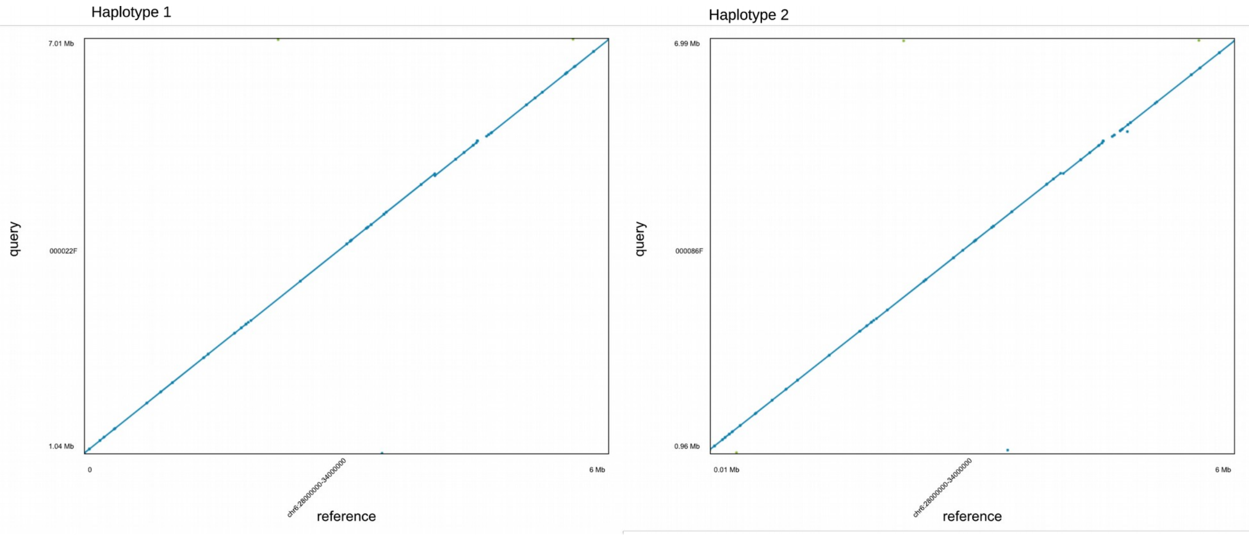

Supplementary Figure 11: Dotplots of the major histocompatibility complex (MHC).

In each haplotype assembly, the whole MHC region is traversed by one single contig. Above we show a corresponding dotplot of the two haplotypes: H1 (left) and H2 (right).

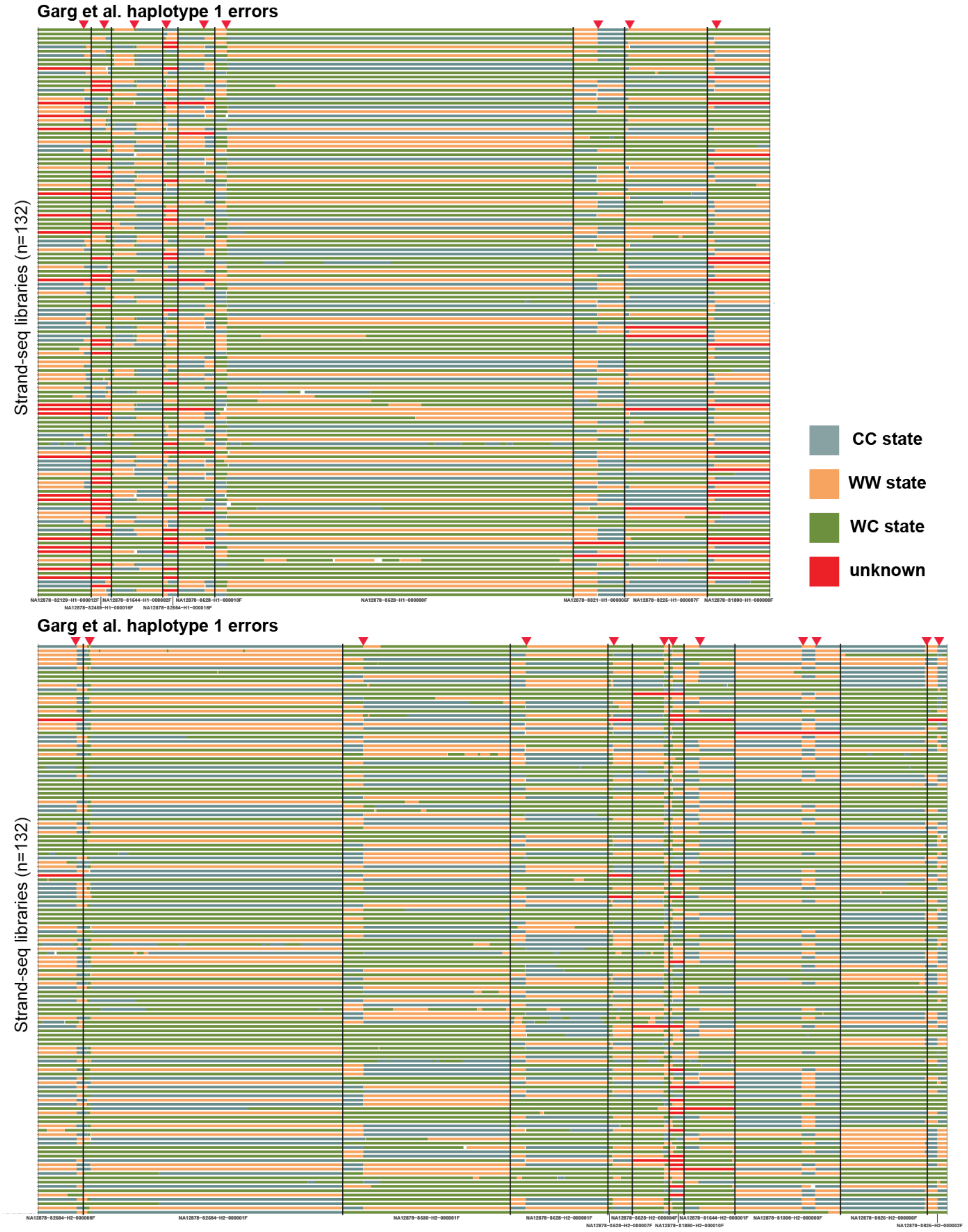

Supplementary Figure 12: Misassembled contigs detected in Garg et al. (2019).
Each row represents a single Strand-seq library (n=132) and colored bars along each line assign each region one of the three possible strand states (WW - only Watson reads, CC - only Crick reads, or WC - mixture of Watson and Crick reads mapped in a given region). Red bars represent regions where genotyping could not be reliably

determined. Black vertical lines delineate separate contigs (not concatenated). Red arrowheads on top of each plot marks the recurrent strand state changes that points to an error in the contig assembly (**Supplementary Fig. 6**).

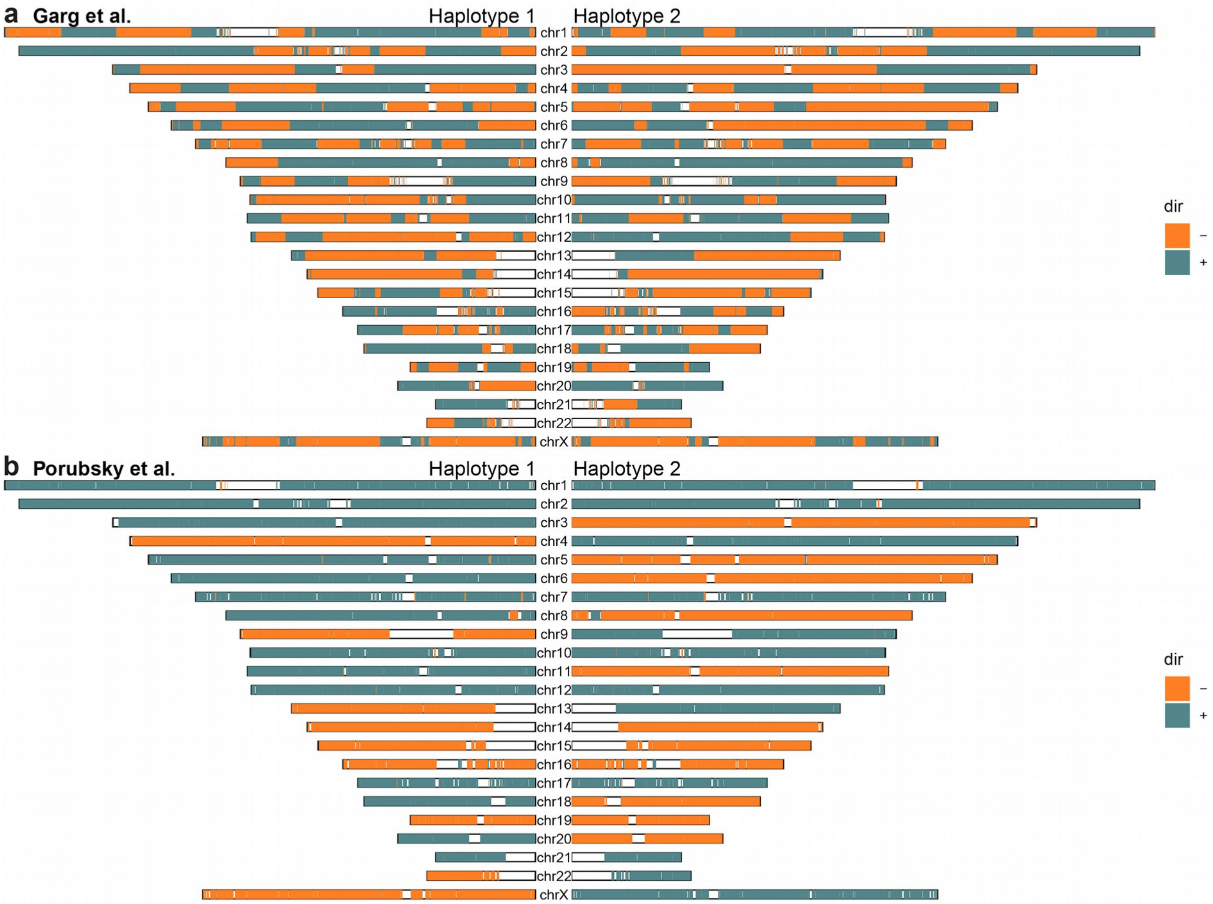

Supplementary Figure 13: Orientation of NA12878-specific contigs in respect to GRCh38.

a) Phased contigs assembled by the WHdenovo pipeline aligned to GRCh38. Each contig represents a range based on mapping coordinates on GRCh38. Contigs are colored based on the directionality ('+' - positive strand, '-' - negative strand) they map to GRCh38. **b**) Phased contigs assembled by our pipeline aligned to GRCh38. Here we observe a single color for each chromosome, which means that contigs that belong to the same chromosome have the same orientation.

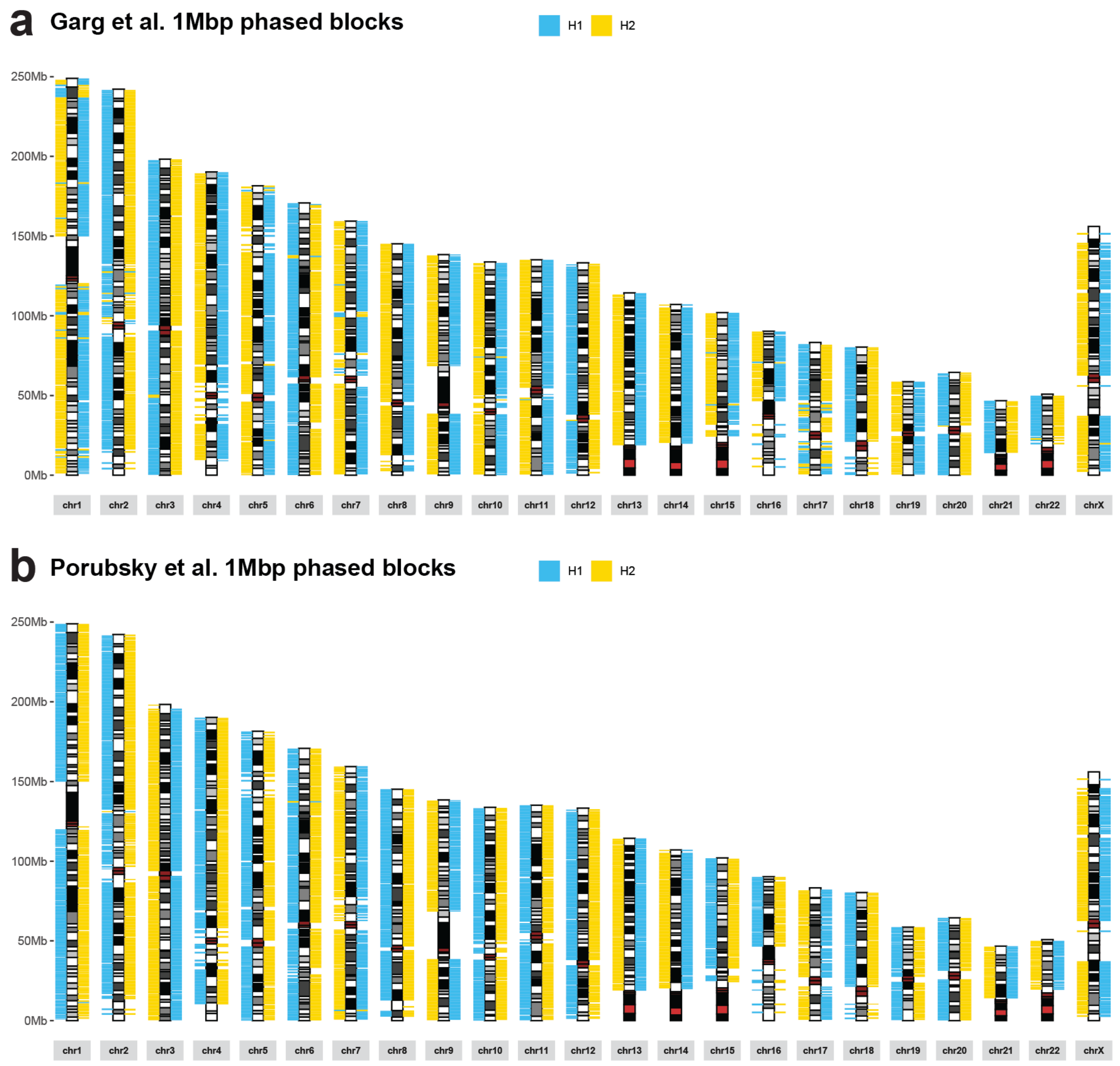

Supplementary Figure 14: Phasing accuracy of NA12878 phased assemblies.

Each 1 Mbp block of phased contigs are assigned to one of the parental genomes using SNV data from the parents (Zook et al. 2014). NA12878 haplotypes are shown to the left (haplotype 1) and to the right (haplotype 2) from each chromosomal ideogram.

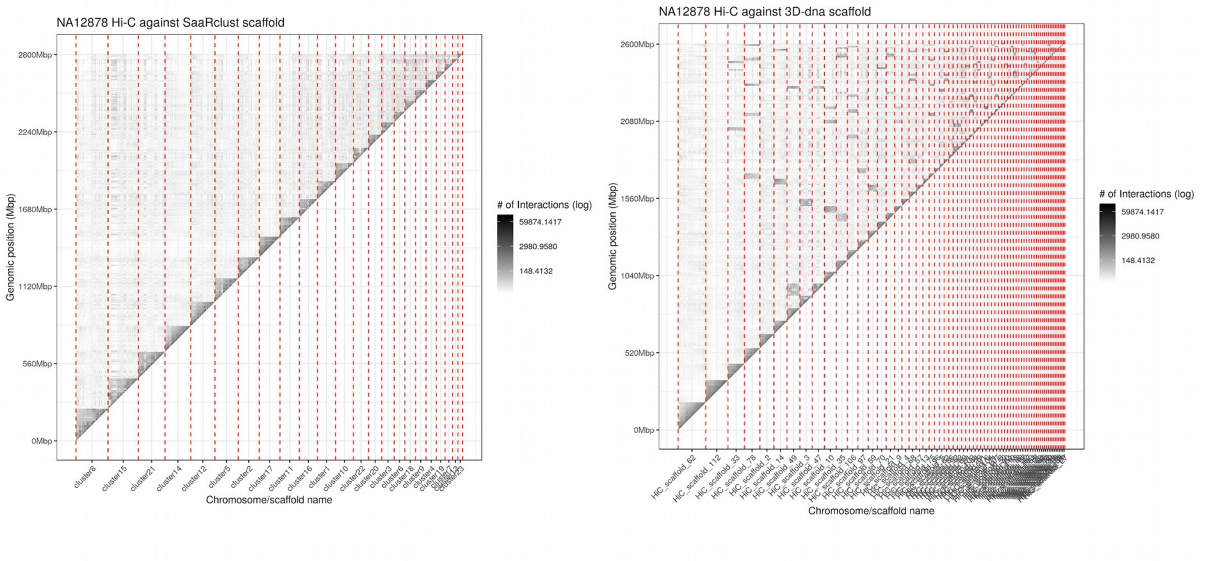

Supplementary Figure 15: Hi-C scaffolding performance of NA12878.

A Hi-C contact matrix constructed from publicly available Hi-C data for NA12878 (**Data availability**) aligned to the SaaRclust and 3D-dna (Dudchenko et al. 2017) based chromosomal scaffolds made from squashed assemblies.

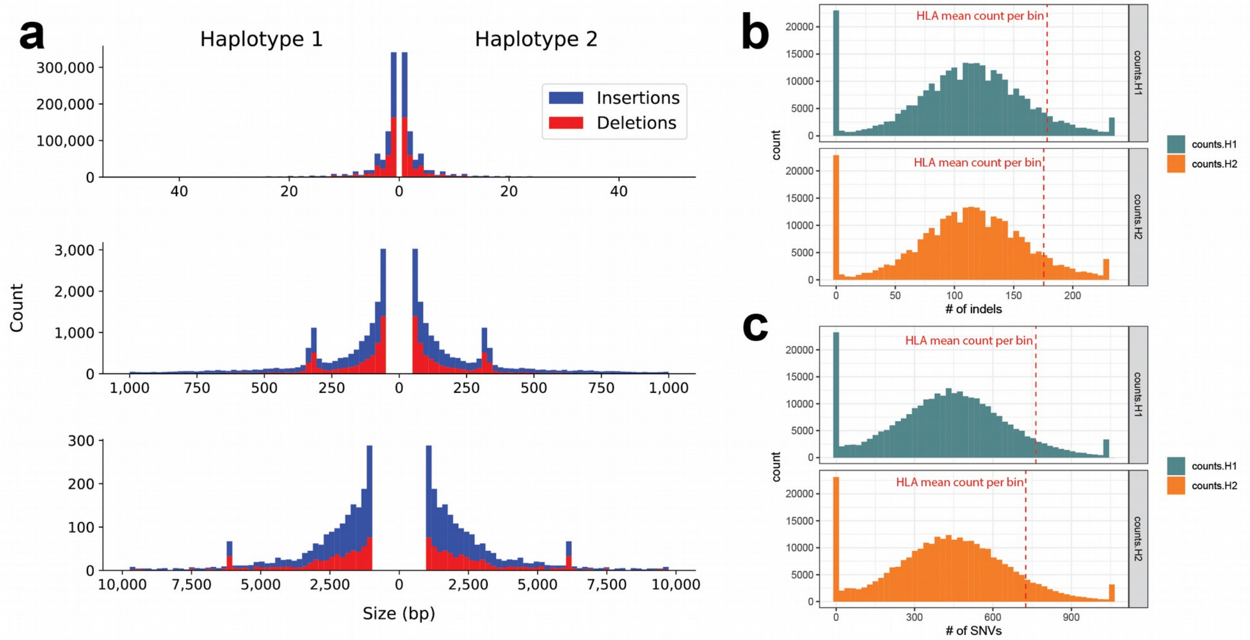

Supplementary Figure 16: Indels density in phased assemblies.

a) Top: Indel (1-49 bp) frequency distribution. Single base-pair indels are most common with peaks at modulo-2 bp events (4, 6, 8, etc.) corresponding to the prevalence of dinucleotide repeat elements. Middle: Smaller SVs (50-999 bp) show a peak for SINE elements. Bottom: Larger SVs show a peak for LINE elements. SVs larger than 10,000 are not shown. **b**) and **c**) Histograms showing the distribution of small indels (b) and SNVs (c) counted in 200 kbp long nonoverlapping bins separately for haplotype 1 (H1 - teal) and haplotype 2 (H2 - orange). Mean indel and SNV counts in bins spanning the HLA locus are highlighted by red dashed lines. Indels and SNVs in regions of detected assembly collapses and known SDs have been removed.

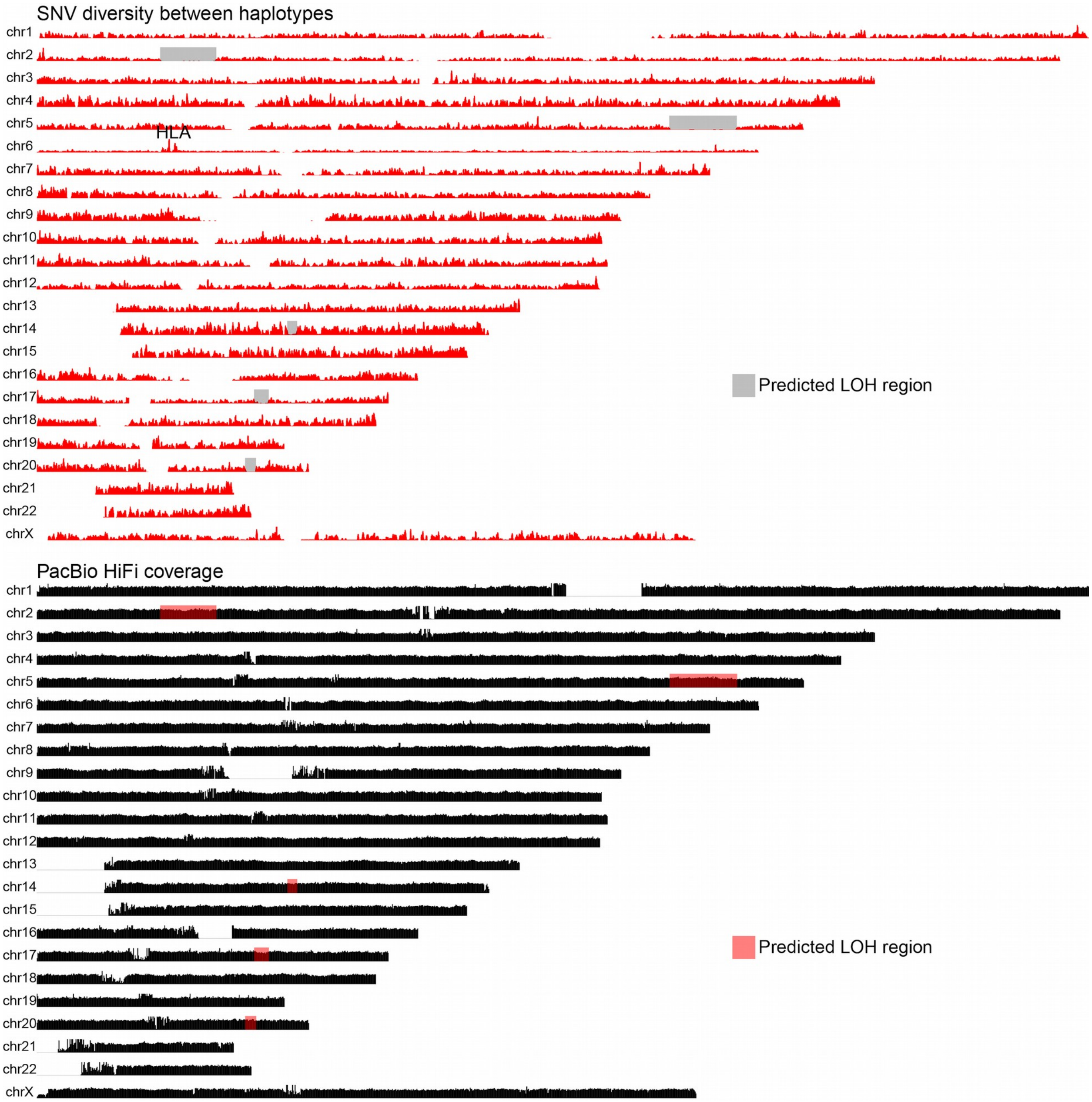

Supplementary Figure 17: Extended regions of homozygosity in HG00733.

Top ideogram: An ideogram showing the diversity between assemblies for H1 and H2. Red vertical bars along each chromosome represent a fraction of heterozygous alleles in 200 kbp bins (sliding by 10 kbp). Bottom ideogram: An ideogram is visualizing HiFi PacBio reads aligned to GRCh38. Black vertical bars along each chromosome represent a number of PacBio reads (mapq >= 10) counted in 200 kbp long bins.

List of detected loss-of-heterozygosity (LOH) regions highlighted in the top and bottom ideogram: chr2:29220001-42500000, chr5:149660001-165620000, chr14:59290001-61610000, chr17:51440001-54890000, chr20:49300001-51910000

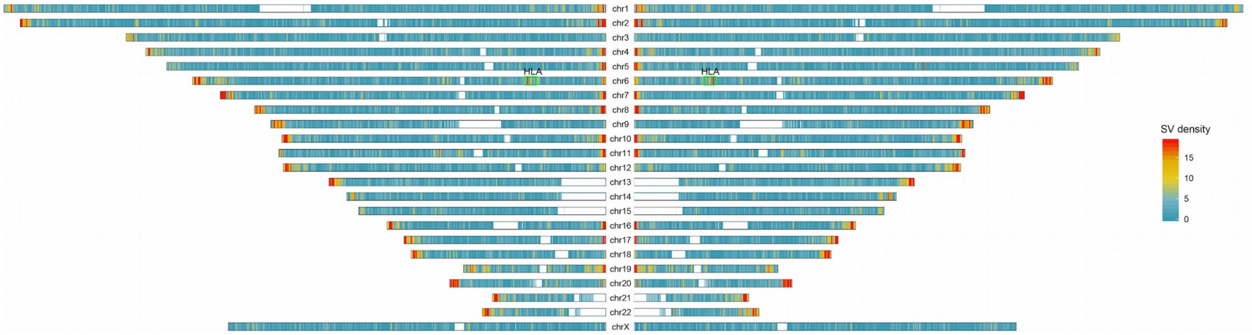

Supplementary Figure 18: Genome-wide distribution of SVs for HG00733.

Genome-wide summary of SV density (>50 bp) counted in 500 kbp genomic bins sliding by 10 kbp. The HLA locus on chromosome 6 is labeled as “HLA”.

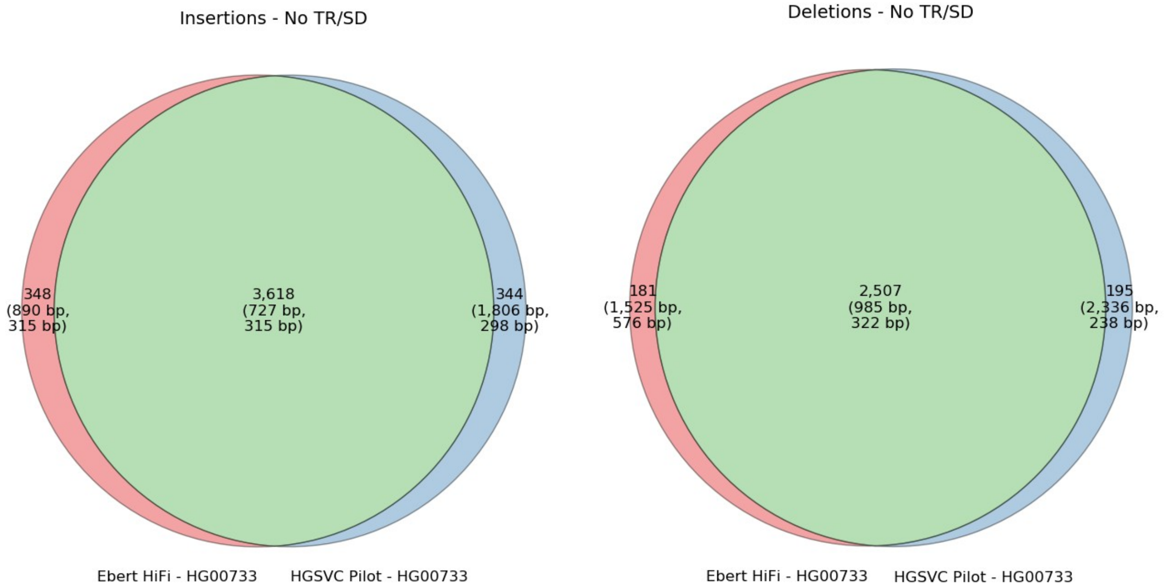

Supplementary Figure 19: Variant comparisons between assembly SVs and HGSVC HG00733 outside tandem repeat (TR) and segmental duplication (SD) loci.

Variant comparisons outside TRs and SDs give a picture of concordance without many of the alignment problems that make repeats difficult to represent and reproduce. The number of variants is shown with the mean (top) and median (bottom) SV size in parentheses. Unplaced and unlocalized SVs were removed from this analysis, which were filtered in HGSVC.

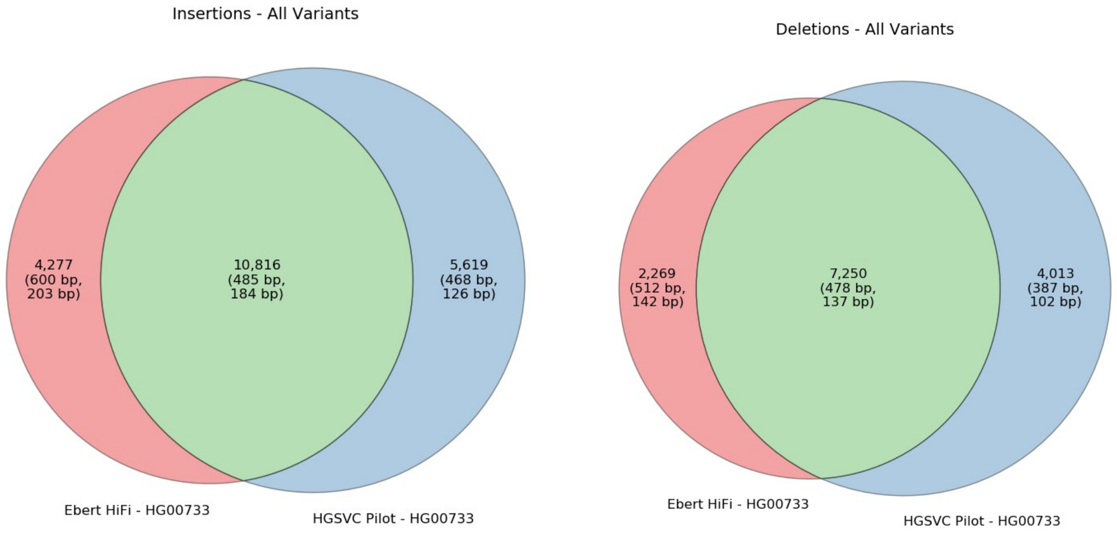

Supplementary Figure 20: Variant comparisons between assembly SVs and HGSVC HG00733.

Variant comparisons including TRs and SDs are harder to replicate, even for larger events, which are often fragmented or shifted by alignments through repeats. The number of variants is shown with the mean (top) and median (bottom) SV size in parentheses. Unplaced and unlocalized SVs were removed from this analysis, which were filtered in HGSVC.

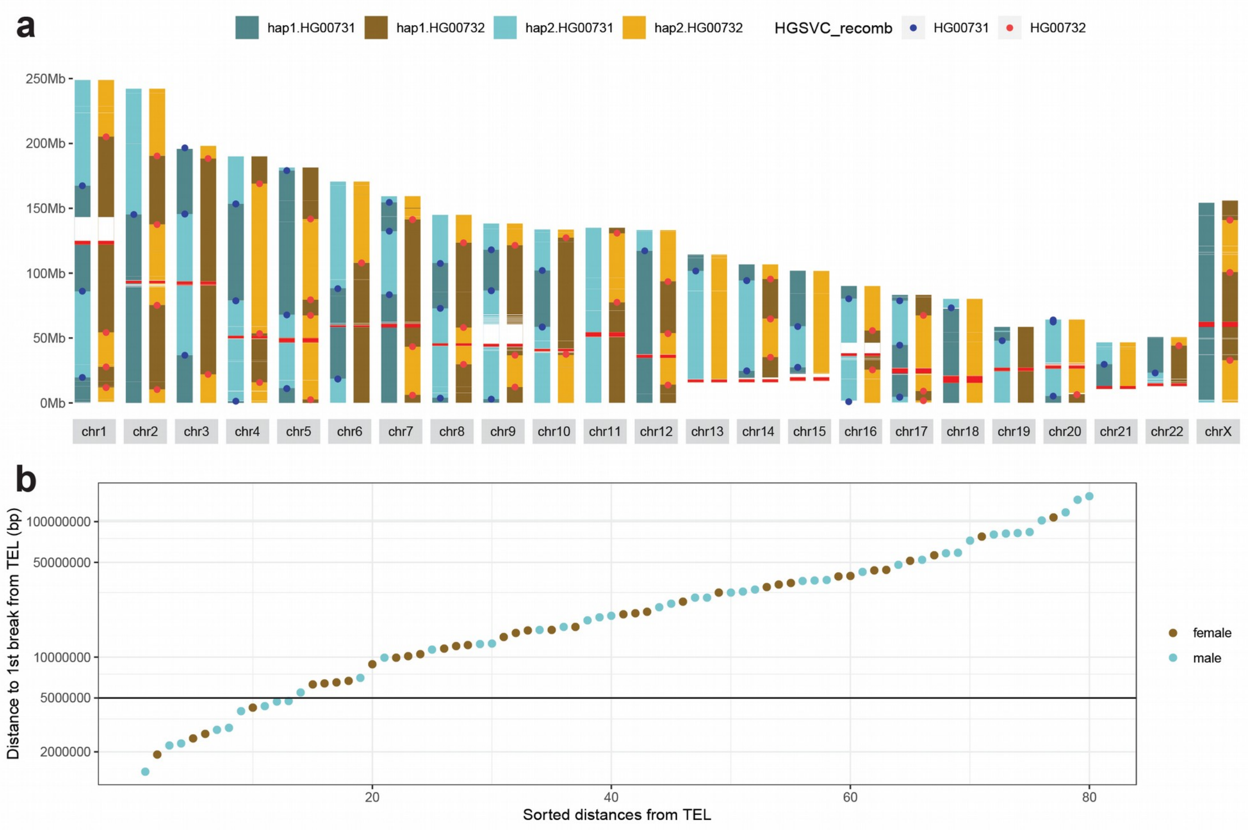

Supplementary Figure 21: Meiotic recombination map of the Puerto Rican trio.
a) Meiotic recombination map for each chromosome shows inherited parts of paternal (paternal homologue H1 - teal, paternal homologue H2 - cyan) and maternal (maternal homologue H1 - brown, maternal homologue H2 - yellow) homologues in the child (HG00733). Assembly gaps are colored in white and centromeres in red. Previously defined meiotic recombination breakpoints (Chaisson et al. 2019) are plotted as dots (blue - paternal (HG00731), red - maternal (HG00732)) over our recombination map. **b**) Sorted distribution of distances of each telomere to the closest meiotic recombination breakpoint specific to female (brown) and male (cyan).

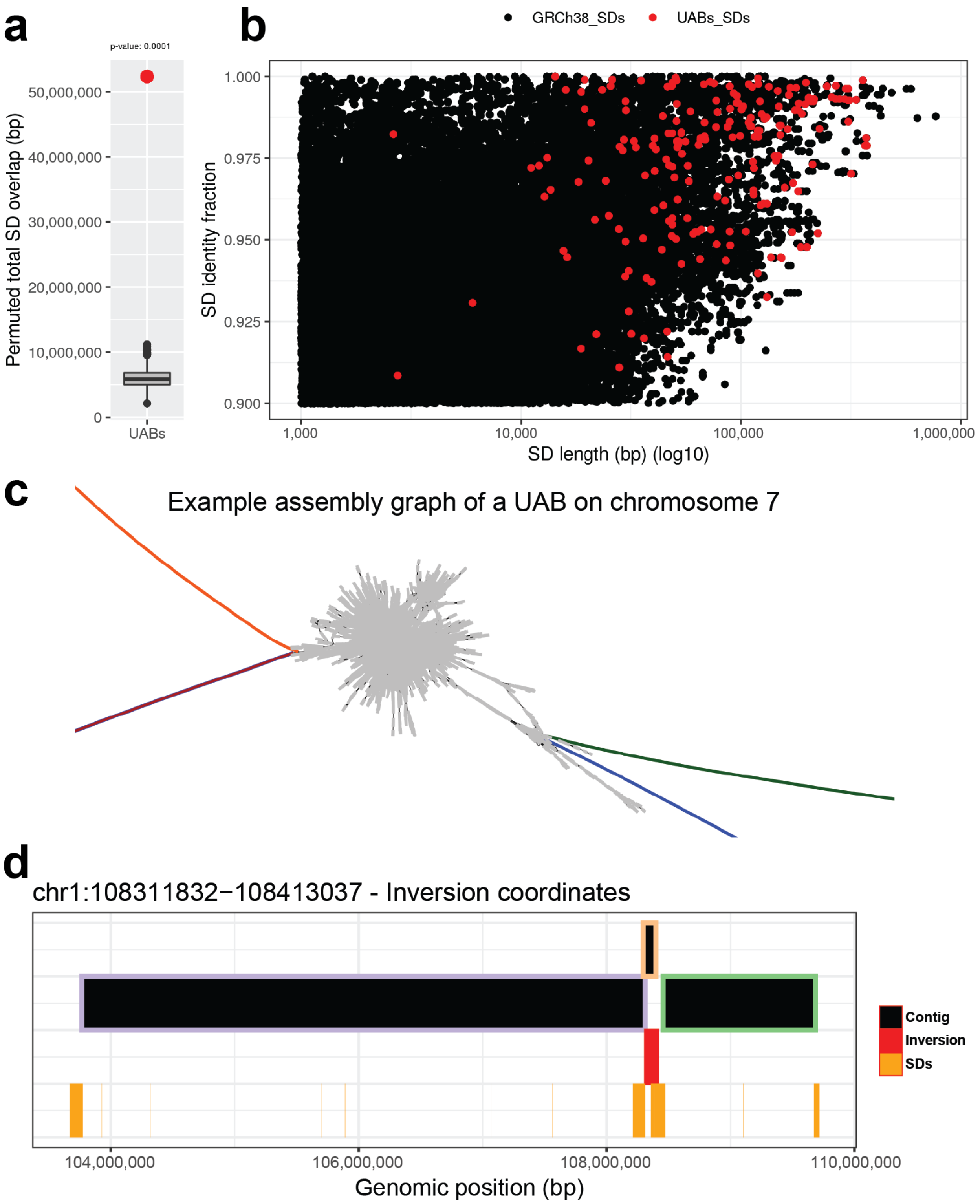

Supplementary Figure 22: Genomic characteristics of UABs.

a) A boxplot that calculates enrichment of total SD base pairs within detected UABs (n=222) in comparison to randomly permuted UABs per chromosome (10,000 permutations) using regioneR package (Gel et al. 2016). **b**) A scatterplot of showing distribution of length and fraction identity of all GRCh38 SDs (black points). For each UAB (n=222) we report the longest SD that overlaps with a UAB and plot them over the original scatterplot in red. **c**) This

graph corresponds to a UAB on chromosome 7 between positions 45,788,351 and 45,828,535 (40,185 base length) for Haplotype 1 (H1). Contig cluster1_000008F (in green) maps to coordinates before the UAB, while contig cluster1_000004F (in blue) is located after the UAB. These contigs are connected by reads (in gray) in the overlap graph. Additionally, the reads connect to two other contigs (cluster1_000002F in brown and cluster1_000000F in orange), creating a topology difficult to resolve by assembly tools and hence breaking the assembly. **d**) An inverted region is plotted in red, while contigs are plotted in black rectangles with colored linings to distinguish discontinuously assembled regions. The SD track is visualized in orange rectangles.

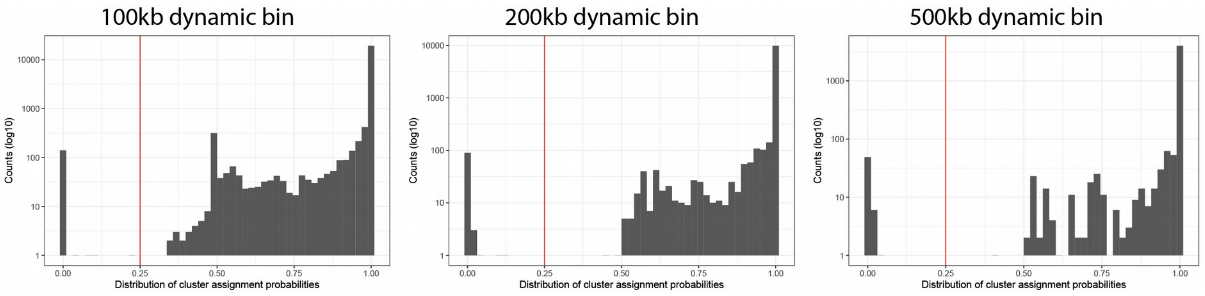

Supplementary Figure 23: Effect of binning strategy on final cluster assignment probabilities.

In SaaRclust, each piece of DNA (contig) is assigned a probability of belonging to any of the tested chromosomal clusters. This probability is calculated using the previously published expectation maximization algorithm (Ghareghani et al. 2018). As expected, some contigs are difficult to assign unambiguously to a single cluster and could be assigned to several clusters with equally low probabilities (left part of the distribution in panels). We examined the effect of varying the bin size from 100 kbp to 500 kbp on the resulting probability distribution. Given the observed probability distribution, we decided to set a dynamic bin size to 200 kbp (SaaRclust ‘bin.size’ parameter) with the probability threshold (SaaRclust ‘prob.th’ parameter) set to 0.25.

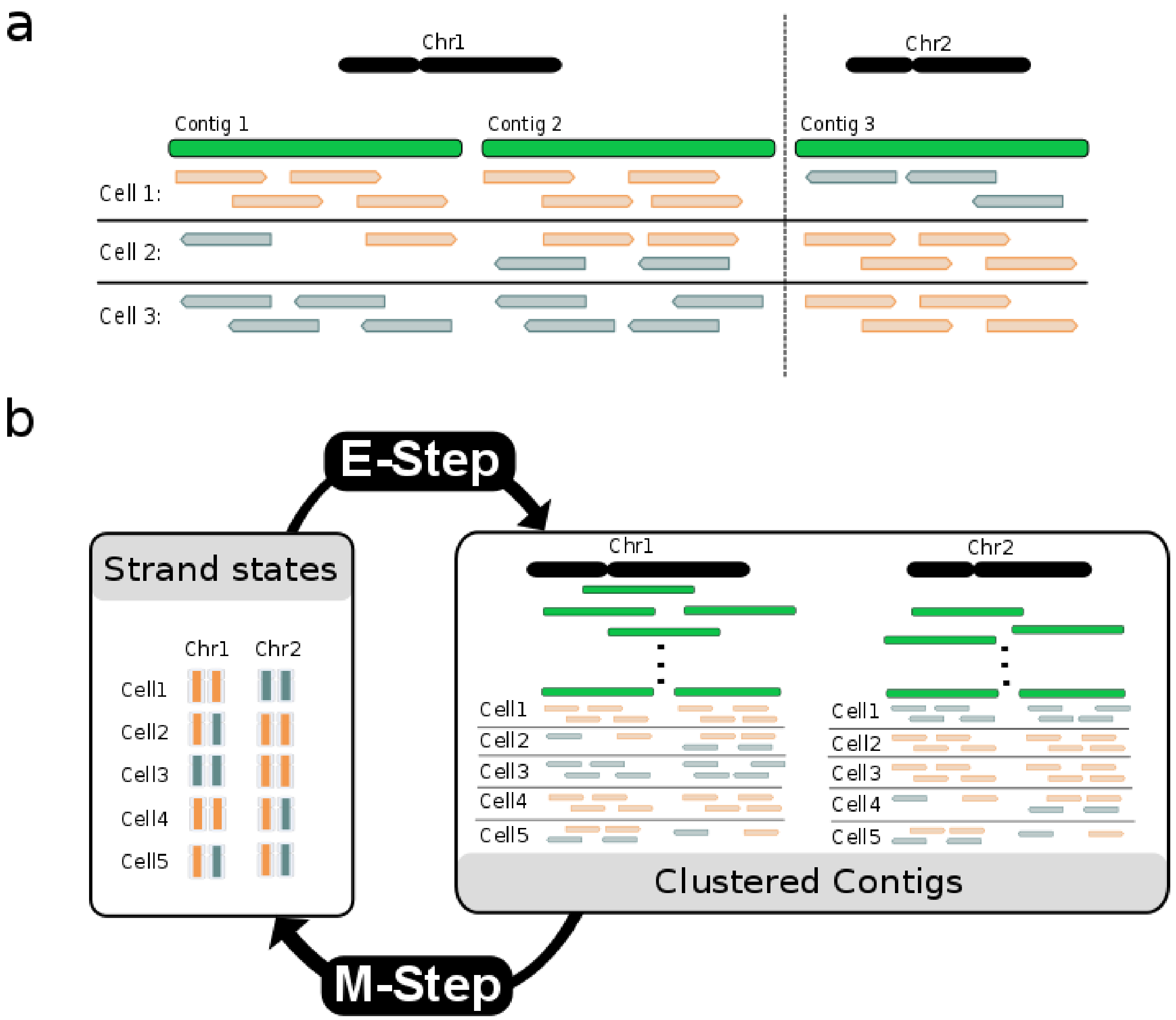

Supplementary Figure 24: An overview of the SaaRclust approach for clustering contigs by chromosome and orientation (Ghareghani et al. 2018)**.**

a) Aligning single-cell Strand-seq reads to squashed contigs. In this example, Strand-seq reads from three different single cells are aligned to three contigs. Strand-seq reads mapped in Watson and Crick directions to contigs are shown by orange and teal colors, respectively. Contigs 1 and 2 come from chromosome 1 showing a different strand state from Contig 3 that comes from chromosome 2. **b**) Schematic of the EM clustering algorithm for two chromosomes. Starting from an arbitrary initialization of strand states, the EM algorithm iterates through the flow of information between the two hidden layers of information: the strand states of single cells in chromosomes (left box) and the clustering of contigs into chromosomes (right box).

Supplementary Tables 1-8

Supplementary Table 1: *De novo* assembly statistics (external xlsx file).

Columns B-F: basic characteristics for each phased assembly (column A). Column F: the contig N50 value is taken from QUAST-LG analysis reports. Columns G-H: switch error and Hamming distance computed as described in the **Methods** section. Columns J-K: Illumina-based QV estimates (**Methods**) counting only HOM SNV (column J) or all HOM variant calls (column K) as errors in the phased assembly. The right QV estimate is computed based only on variants in high-confidence regions (**Methods**), the left number additionally takes variant calls into account that were not lifted to the GRCh38 reference. Column L: parameter set used to generate the respective assembly; see the pipeline repository (**Code availability**). Column M: FASTA file name of phased assembly (**Data availability**).

Supplementary Table 2: Comparison of our phased assembly pipeline with Hi-C-based assemblies.

***analysis done with only 10 BACs**
****these values were calculated for corrected assemblies by SaaRclust, marked as *_corr in Supplementary Table 1.**

	Sample	Contig N50 (Mbp)		Assembly size (Gbp)		BAC QV	Short-read based QV		Switch error rate		Hamming Distance		Assembly errors**		Contig directionality**	
		H1	H2	H1	H2		H1	H2	H1	H2	H1	H2	H1	H2	H1	H2
Garg et al.	NA12878	19.9	18.9	2.97	2.97	42.382067	48-57	48-57	0.836%	0.842%	1.487%	1.487%	9	11	65.29%	67.38%
Porubsky et al.	NA12878	18.3	21.9	2.88	2.88	42.059018	51-60	51-60	0.449%	0.435%	0.406%	0.395%	3	1	99.53%	99.55%
FALCON-phase	HG00733	26.3	26.3	2.89	2.89	36.027976*	38-38	38-38	0.781%	0.782%	36.87%	36.90%	10	10	63.61%	63.63%
Porubsky et al.	HG00733	23.7	25.9	2.92	2.92	40.467447*	50-59	51-59	0.169%	0.171%	0.167%	0.168%	2	2	99.80%	99.66%

Supplementary Table 3: Phased assembly indel discovery.

Indels were discovered in both haplotypes and merged into a single call. Fields are number of variants (“N”), mean indel size (“Mean (bp)”), total number of indel bases (“Base (kbp)”), the percentage 1 bp indels (“1 bp (%)”), and the percentage heterozygous calls (“Het (%)”).

	Insertions					Deletions				
Assembly	N	Mean (bp)	Base (kbp)	1 bp (%)	Het (%)	N	Mean (bp)	Base (kbp)	1 bp (%)	Het (%)
HG00733 (Racon x2)	510,393	3.42	1,747	50.03%	60.50%	494,225	3.69	1,826	47.56%	61.58%
HG00733 (unpolished)	513,105	3.44	1,765	50.47%	62.14%	528,439	3.58	1,894	49.23%	63.06%

Supplementary Table 4: Phased assembly structural variation discovery.

Variants were discovered in both haplotypes and merged to a set of homozygous and heterozygous calls. Fields are number of variants (“N”), mean variant size (“Mean (bp)”), sum of all variant lengths (“Base (Mbp)”), and the percentage of heterozygous calls by number (“Het (N%)”) and by bases (“Het (bp %)”).

	Insertions	Deletions

Assembly	N	Mean (bp)	Base (Mbp)	Het (N%)	Het (bp %)	N	Mean (bp)	Tput (Mbp)	Het (N%)	Het (bp %)
HG00733 (Racon x2)	15,093	517	7.80	59.27%	52.37%	9,519	485	4.62	65.82%	66.03%
HG00733 (unpolished)	15,175	515	7.81	59.35%	52.51%	9,523	481	4.58	65.79%	65.77%

Supplementary Table 5: Frameshift-disrupted RefSeq annotations.

We quantified the number of genes with a frameshift indel or SV in coding regions and demonstrate that polishing is still required for phased Peregrine assemblies. Shown are disrupted gene counts for all genes (“All”), genes with no exons intersecting tandem repeats or segmental duplications (“No TR/SD”), and genes with at least one exon in a known segmental duplication (“In SD”).

Sample	Assembler	Polishing	All	No TR/SD	In SD
HG00733	Peregrine	Racon x2	223	88	68
HG00733	Peregrine	None	301	110	112

Supplementary Table 6: List of detected UABs (external xlsx file).

Supplementary Table 7: HiFi PacBio sequencing summary.

**Sample**	**HG00731**	**HG00732**	**HG00733**
**# SMRT Cell 8Ms**	5	6	7
**Raw Base Yield (Gbp)**	1612	1138	1568
**HiFi Base Yield (Gbp)**	103	67	104
**HiFi Coverage (X)**	32	21	32
**Average HiFi Read Length (kbp)**	11.1	10.7	13.6
**Median HiFi QV**	31.86	31.59	30.39
**Average HiFi number of passes**	10.51	10.54	9.34

Supplementary Table 8: Accession IDs to data used in this study (external xlsx file).

Human Genome Structural Variation Consortium

Steering Committee: Evan E. Eichler, Jan O. Korbel, Charles Lee
Consortium Members (alphabetical order): Haley Abel, Alexej Abyzov, Can Alkan, Thomas Anantharaman, Danny Antaki, Peter A. Audano, Ali Bashir, Mark Batzer, Harrison Brand, Lisa Brooks, Stuart Cantsilieris, Han Cao, Eliza Cerveira, Mark J. P. Chaisson, Ken Chen, Chong Chen, Xintong Chen, Chen-Shan Chin, Zechen Chong, Nelson T. Chuang, Deanna M. Church, Laura Clarke, Ryan L. Collins, Robel Dagnow, Scott E. Devine, Li Ding, Peter Ebert, Susan Fairley, Xian Fan, Andrew Farrell, Ian Fiddes, Paul Flicek, Joey Flores, Daniel Fordham, Timur Galeev, Eugene J. Gardner, Mark B. Gerstein, David U. Gorkin, Madhusudan Gujral, Li Guo, Gamze Gursoy, Victor Guryev, Ira Hall, Robert E. Handsaker, Eoghan Harrington, William Harvey, Alex R. Hastie, William Haynes Heaton, Wolfram Hoeps, Fereydoun Hormozdiari, Junie Jen, Goo Jun, Chong Lek Koh, Xiangmeng Kong, Miriam Konkel, Jonas Korlach, Zev N. Kronenberg, Sushant Kumar, Pui-Yan Kwok, Jee Young Kwon, Sofia Kyriazopoulou-Panagiotopoulou, Ernest T. Lam, Christine C. Lambert, Peter M. Lansdorp, Jong Eun Lee, Sau Peng Lee, Wan-Ping Lee, Dillon Lee, Joyce Lee, Shantao Li, Ernesto Lowy Gallego, Shamoni Maheshwari, Ankit Malhotra, Patrick Marks, Tobias Marschall, Gabor T. Marth, Alvaro Martinez Barrio, Adam Mattson, Steven McCarroll, Sascha Meiers, Ryan E. Mills, Katherine M. Munson, Fabio C. P. Navarro, Bradley J. Nelson, Conor Nodzak, Amina Noor, Andy W. C. Pang, David Porubsky, Letu Qingge, Yunjiang Qiu, Tobias Rausch, Allison Regier, Bing Ren, Oscar L. Rodriguez, Gabriel Rosanio, Joel Rozowsky, Mallory Ryan, Ashley D. Sanders, Michael Schnall-Levin, Jonathan Sebat, Omar Shanta, Steve Sherry, Xinghua Shi, Laura Carolyn Smith, Mike Smith, Diana C. J. Spierings, Adrian Stütz, Arvis Sulovari, Michael E. Talkowski, Karine Viaud-Martinez, Alistair Ward, Anne Marie E. Welch, Jia Wen, Aaron M. Wenger, Matthew Wyczalkowski, Ming Xiao, Wei Xu, Sergei Yakneen, Xiaofei Yang, Kai Ye, Christopher Yoon, Chengsheng Zhang, Xuefang Zhao, Xiangqun Zheng-Bradley, Arthur Zhou, Qihui Zhu, Mike Zody

References

Audano, Peter A., Arvis Sulovari, Tina A. Graves-Lindsay, Stuart Cantsilieris, Melanie Sorensen, Annemarie E. Welch, Max L. Dougherty, et al. 2019. “Characterizing the Major Structural Variant Alleles of the Human Genome.” *Cell* 176: 1–13.

Chaisson, Mark J. P., Ashley D. Sanders, Xuefang Zhao, Ankit Malhotra, David Porubsky, Tobias Rausch, Eugene J. Gardner, et al. 2019. “Multi-Platform Discovery of Haplotype-Resolved Structural Variation in Human Genomes.” *Nature Communications* 10 (1): 1784.

Claussin, Clémence, David Porubský, Diana Cj Spierings, Nancy Halsema, Stefan Rentas, Victor Guryev, Peter M. Lansdorp, and Michael Chang. 2017. “Genome-Wide Mapping of Sister Chromatid Exchange Events in Single Yeast Cells Using Strand-Seq.” *eLife* 6 (December). https://doi.org/10.7554/eLife.30560.

Dudchenko, Olga, Sanjit S. Batra, Arina D. Omer, Sarah K. Nyquist, Marie Hoeger, Neva C. Durand, Muhammad S. Shamim, et al. 2017. “De Novo Assembly of the Aedes Aegypti Genome Using Hi-C Yields Chromosome-Length Scaffolds.” *Science* 356 (6333): 92–95.

Falconer, Ester, Mark Hills, Ulrike Naumann, Steven S. S. Poon, Elizabeth A. Chavez, Ashley D. Sanders, Yongjun Zhao, Martin Hirst, and Peter M. Lansdorp. 2012. “DNA Template Strand Sequencing of Single-Cells Maps Genomic Rearrangements at High Resolution.” *Nature Methods* 9 (11): 1107–12.

Gel, Bernat, Anna Díez-Villanueva, Eduard Serra, Marcus Buschbeck, Miguel A. Peinado, and Roberto Malinverni. 2016. “regioneR: An R/Bioconductor Package for the Association Analysis of Genomic Regions Based on Permutation Tests.” *Bioinformatics* 32 (2): 289–91.

Ghareghani, Maryam, David Porubsk, Ashley D. Sanders, Sascha Meiers, Evan E. Eichler, Jan O. Korbel, and Tobias Marschall. 2018. “Strand-Seq Enables Reliable Separation of Long Reads by Chromosome via Expectation Maximization.” *Bioinformatics* 34 (13): i115–23.

Hills, Mark, Kieran O’Neill, Ester Falconer, Ryan Brinkman, and Peter M. Lansdorp. 2013. “BAIT: Organizing Genomes and Mapping Rearrangements in Single Cells.” *Genome Medicine* 5 (9): 82.

Huddleston, John, Mark J. P. Chaisson, Karyn Meltz Steinberg, Wes Warren, Kendra Hoekzema, David Gordon, Tina A. Graves-Lindsay, et al. 2017. “Discovery and Genotyping of Structural Variation from Long-Read Haploid Genome Sequence Data.” *Genome Research* 27 (5): 677–85.

Jauch, A., J. Wienberg, R. Stanyon, N. Arnold, S. Tofanelli, T. Ishida, and T. Cremer. 1992. “Reconstruction of Genomic Rearrangements in Great Apes and Gibbons by Chromosome Painting.” *Proceedings of the National Academy of Sciences of the United States of America* 89 (18): 8611–15.

Li, Heng. 2018. “Minimap2: Pairwise Alignment for Nucleotide Sequences.” *Bioinformatics* , May. https://doi.org/10.1093/bioinformatics/bty191.

Marijon, Pierre, Rayan Chikhi, and Jean-Stéphane Varré. 2019a. “Yacrd and Fpa: Upstream Tools for Long-Read Genome Assembly.” *bioRxiv*. https://doi.org/10.1101/674036.

———. 2019b. “Graph Analysis of Fragmented Long-Read Bacterial Genome Assemblies.” *Bioinformatics* 35 (21): 4239–46.

Porubsky, David, Shilpa Garg, Ashley D. Sanders, Jan O. Korbel, Victor Guryev, Peter M. Lansdorp, and Tobias Marschall. 2017. “Dense and Accurate Whole-Chromosome Haplotyping of Individual Genomes.” *Nature Communications* 8 (1): 1293.

Porubský, David, Ashley D. Sanders, Niek van Wietmarschen, Ester Falconer, Mark Hills, Diana C. J. Spierings, Marianna R. Bevova, Victor Guryev, and Peter M. Lansdorp. 2016. “Direct Chromosome-Length Haplotyping by Single-Cell Sequencing.” *Genome Research* 26 (11): 1565–74.

Sanders, Ashley D., Ester Falconer, Mark Hills, Diana C. J. Spierings, and Peter M. Lansdorp. 2017. “Single-Cell Template Strand Sequencing by Strand-Seq Enables the Characterization of Individual Homologs.” *Nature Protocols* 12 (6): 1151–76.

Sanders, Ashley D., Mark Hills, David Porubský, Victor Guryev, Ester Falconer, and Peter M. Lansdorp. 2016. “Characterizing Polymorphic Inversions in Human Genomes by Single-Cell Sequencing.” *Genome Research* 26 (11): 1575–87.

Sanders, Ashley D., Sascha Meiers, Maryam Ghareghani, David Porubsky, Hyobin Jeong, M. Alexandra C. C. van Vliet, Tobias Rausch, et al. 2019. “Single-Cell Analysis of Structural Variations and Complex Rearrangements with Tri-Channel Processing.” *Nature Biotechnology*, December. https://doi.org/10.1038/s41587-019-0366-x.

Stankiewicz, Paweł, Christine J. Shaw, Marjorie Withers, Ken Inoue, and James R. Lupski. 2004. “Serial Segmental Duplications during Primate Evolution Result in Complex Human Genome Architecture.” *Genome Research* 14 (11): 2209–20.

Wick, Ryan R., Mark B. Schultz, Justin Zobel, and Kathryn E. Holt. 2015. “Bandage: Interactive Visualization of de Novo Genome Assemblies.” *Bioinformatics* 31 (20): 3350–52.

Wietmarschen, Niek van, and Peter M. Lansdorp. 2016. “Bromodeoxyuridine Does Not Contribute to Sister Chromatid Exchange Events in Normal or Bloom Syndrome Cells.” *Nucleic Acids Research* 44 (14): 6787–93.

Zook, Justin M., Brad Chapman, Jason Wang, David Mittelman, Oliver Hofmann, Winston Hide, and Marc Salit. 2014. “Integrating Human Sequence Data Sets Provides a Resource of Benchmark SNP and Indel Genotype Calls.” *Nature Biotechnology* 32 (3): 246–51.
