# Supplementary material for: Fully phased human genome assembly without parental data using single-cell strand sequencing and long reads
Source: Nat Biotechnol. 2020 Dec 7;39(3):302–8. doi: 10.1038/s41587-020-0719-5 (PMC7954704; doi:10.1038/s41587-020-0719-5)
Supplement: Supplementary file 2 — Reporting Summary [file 41587_2020_719_MOESM2_ESM.pdf]

natureresearch

Corresponding author(s): Evan Eichler and Tobias Marschall

Last updated by author(s): Jul 24, 2020

Reporting Summary

Nature Research wishes to improve the reproducibility of the work that we publish. This form provides structure for consistency and transparency in reporting. For further information on Nature Research policies, see Authors & Referees and the Editorial Policy Checklist.

Statistics

For all statistical analyses, confirm that the following items are present in the figure legend, table legend, main text, or Methods section.

n/a	Confirmed	
☒	☐	The exact sample size (*n*) for each experimental group/condition, given as a discrete number and unit of measurement
☒	☐	A statement on whether measurements were taken from distinct samples or whether the same sample was measured repeatedly
☒	☐	The statistical test(s) used AND whether they are one- or two-sided<br>*Only common tests should be described solely by name; describe more complex techniques in the Methods section.*
☒	☐	A description of all covariates tested
☒	☐	A description of any assumptions or corrections, such as tests of normality and adjustment for multiple comparisons
☒	☐	A full description of the statistical parameters including central tendency (e.g. means) or other basic estimates (e.g. regression coefficient) AND variation (e.g. standard deviation) or associated estimates of uncertainty (e.g. confidence intervals)
☒	☐	For null hypothesis testing, the test statistic (e.g. *F*, *t*, *r*) with confidence intervals, effect sizes, degrees of freedom and *P* value noted<br>*Give P values as exact values whenever suitable.*
☒	☐	For Bayesian analysis, information on the choice of priors and Markov chain Monte Carlo settings
☒	☐	For hierarchical and complex designs, identification of the appropriate level for tests and full reporting of outcomes
☒	☐	Estimates of effect sizes (e.g. Cohen's *d*, Pearson's *r*), indicating how they were calculated

Our web collection on statistics for biologists contains articles on many of the points above.

Software and code

Policy information about availability of computer code

Data collection	Only sequencing data was analyzed (Illumina/Pacific Biosciences), so no specific software for data collection was used.
Data analysis	Software and workflows developed/adapted for this study (SaaRclust, breakpointR, StrandPhaseR, Snakemake Workflow) are all available under MIT license from the respective public repositories (https://github.com/daewoooo/SaaRclust, https://bioconductor.org/packages/breakpointR, https://github.com/daewoooo/StrandPhaseR devel branch,https://github.com/ptrebert/project-diploid-assembly). For the presented analyses, the v0.1dev has been used (https://github.com/ptrebert/project-diploid-assembly/tree/v0.1dev).

For manuscripts utilizing custom algorithms or software that are central to the research but not yet described in published literature, software must be made available to editors/reviewers. We strongly encourage code deposition in a community repository (e.g. GitHub). See the Nature Research guidelines for submitting code & software for further information.

Data

Policy information about availability of data

All manuscripts must include a data availability statement. This statement should provide the following information, where applicable:

- Accession codes, unique identifiers, or web links for publicly available datasets
- A list of figures that have associated raw data
- A description of any restrictions on data availability

HiFi PacBio reads for HG00731, HG00732, and HG00733 were produced as part of this study and are available from the IGSR FTP (ftp.1000genomes.ebi.ac.uk/vol1/ftp/data_collections/HGSVC2/working/20190925_PUR_PacBio_HiFi/). Strand-seq data for HG00733 were downloaded from NCBI SRA (BioProject PRJEB12849). Illumina short reads for HG00733 were downloaded from NCBI SRA (BioProject PRJEB9396). The genome assemblies produced in this study are available from ftp://ftp.1000genomes.ebi.ac.uk/vol1/ftp/data_collections/HGSVC2/working/20200417_Marschall-Eichler_NBT_hap-assm/.

Field-specific reporting

Please select the one below that is the best fit for your research. If you are not sure, read the appropriate sections before making your selection.

☒ Life sciences ☐ Behavioural & social sciences ☐ Ecological, evolutionary & environmental sciences

For a reference copy of the document with all sections, see nature.com/documents/nr-reporting-summary-flat.pdf

Life sciences study design

All studies must disclose on these points even when the disclosure is negative.

Sample size	One genome was selected for assembly. The parent genomes were assembled for quality control.
Data exclusions	No data was excluded
Replication	Not applicable.
Randomization	Not applicable.
Blinding	Not applicable.

Reporting for specific materials, systems and methods

We require information from authors about some types of materials, experimental systems and methods used in many studies. Here, indicate whether each material, system or method listed is relevant to your study. If you are not sure if a list item applies to your research, read the appropriate section before selecting a response.

Materials & experimental systems

n/a	Involved in the study	
☒	☐	Antibodies
☐	☒	Eukaryotic cell lines
☒	☐	Palaeontology
☒	☐	Animals and other organisms
☒	☐	Human research participants
☒	☐	Clinical data

Methods

n/a	Involved in the study	
☒	☐	ChIP-seq
☒	☐	Flow cytometry
☒	☐	MRI-based neuroimaging

Eukaryotic cell lines

Policy information about cell lines

Cell line source(s)	All cell lines used in this study (HG00732, HG00731, HG00733) can be obtained from the Coriell Biorepository.
Authentication	SNV data was compared to previous sequencing data from Chaisson et al. (Nature Communications, 2019), which confirmed the identity of the sample.
Mycoplasma contamination	Cell lines have been tested before shipping by Coriell. We did not perform further in house tests.
Commonly misidentified lines (See ICLAC register)	n/a
